# Impact of correlated PBPK parameters on intra‐patient global sensitivity analysis in molecular radiotherapy

**DOI:** 10.1002/mp.70746

**Published:** 2026-11-02

**Authors:** Elham Yousefzadeh‐Nowshahr, Deni Hardiansyah, Valentina Vasic, Anna‐Lena Theisen, Johannes Tran‐Gia, Philipp E. Hartrampf, Ambros J. Beer, Michael Lassmann, Gerhard Glatting

**Affiliations:** ^1^ Medical Radiation Physics Department of Nuclear Medicine Ulm University Ulm Germany; ^2^ Department of Nuclear Medicine University Hospital Ulm Ulm Germany; ^3^ Medical Physics and Biophysics Physics Department Faculty of Mathematics and Natural Sciences Universitas Indonesia Depok Indonesia; ^4^ Department of Nuclear Medicine University Hospital Würzburg Würzburg Germany

**Keywords:** Dependent PBPK model parameters, Dosimetry, Intra‐patient global sensitivity analysis, Molecular radiotherapy, Uncertainty

## Abstract

**Background:**

Physiologically based pharmacokinetic (PBPK) models integrated into digital twins provide a robust framework for optimizing individualized molecular radiotherapy (MRT). Nevertheless, uncertainties in PBPK‐model‐derived dosimetry can compromise the reliability of dosimetry‐guided treatment planning and individualized therapy decisions.

**Purpose:**

This study employs intra‐patient global sensitivity analysis (GSA) to assess the influence of uncertainty and correlations among PBPK model parameters on estimated kidney and tumor absorbed doses (ADs) in prostate‐specific membrane antigen (PSMA)‐targeted theragnostics.

**Methods:**

Data from 10 patients with metastatic castration‐resistant prostate cancer who underwent pre‐therapeutic [^68^Ga]Ga‐PSMA‐I&T PET/CT and a cycle of [^177^Lu]Lu‐PSMA‐I&T therapy, followed by post‐therapeutic SPECT/CT scans at Days 1 and 4 post‐injection, were analyzed. A previously established whole‐body PBPK model for PSMA‐targeted radionuclide therapy was used as the baseline model. Fifteen model parameters, their standard errors, and inter‐parameter correlations were estimated by fitting to measured time‐activity data and subsequently evaluated using intra‐patient GSA based on the Kucherenko method implemented in UQLab. In one approach, correlations were ignored to identify the direct influences of uncertainty in input parameters on ADs. In a second approach, correlations were included to reflect realistic parameter distributions and to capture indirect effects arising from parameter interdependencies. Pharmacokinetics were simulated to estimate therapeutic kidney and tumor ADs as well as main‐ and total‐effect indices ($S_{i},S_{i}^{T})$. Sensitivity indices, ADs, and their associated uncertainty metrics were compared between scenarios with and without parameter correlations.

**Results:**

Including parameter correlations in the GSA yielded kidney ADs of 4.2 Gy [3.0–7.2 Gy] (median [min–max]) with standard deviations (SDs) of 0.5 Gy [0.4–0.9 Gy] and tumor ADs of 10.1 Gy [2.0–26.8 Gy] with SDs of 1.7 Gy [0.6–3.4 Gy]. Neglecting correlations resulted in significantly higher uncertainties in ADs, by a factor of 1.2 [1.0–1.8] for both kidneys and tumors. Kidney ADs were most sensitive to kidney receptor density ($S_{i}=0.3\;\left[0.1-\;0.6\right],$ $S_{i}^{T}=0.7\;\left[0.6-0.8\right]$) and to the filtered fraction of renal blood flow $(S_{i}=0.2\;\left[0.1-0.3\right],$ $S_{i}^{T}=0.3\;\left[0.1-0.4\right])$. Tumor ADs were most affected by tumor receptor density ($S_{i}=0.7\;\left[0.1-1.0\right],$ $S_{i}^{T}=0.8\;\left[0.3-1.0\right]$). The involvement of parameter correlations did not alter the identification of the parameters most influential on AD estimates and further reduced computational runtime.

**Conclusions:**

Incorporating parameter correlations into intra‐patient GSA reduced the estimated uncertainty associated with ADs, underscoring the importance of preserving parameter dependencies when propagating uncertainty in PBPK‐based individualized MRT. The identification of key PBPK parameters governing kidney and tumor ADs remained stable when including correlation, confirming the robustness of the underlying model structure.

## 1 INTRODUCTION

Molecular radiotherapy (MRT) has achieved significant success in treating various cancer types over the past decade.^1^, ^2^, ^3^, ^4^, ^5^ However, the conventional practice of administering the same fixed activity to all patients does not account for individual patient characteristics and, therefore, cannot be considered an individualized treatment approach. Improved therapeutic outcomes can reasonably be expected when the administered activity is tailored to the individual patient.

Digital twins, which integrate PBPK models with patient‐specific clinical and imaging data, provide a powerful framework for personalized therapy planning.^6^, ^7^, ^8^, ^9^, ^10^, ^11^ They enable the prediction of treatment outcomes and the optimization of the administered activity, thereby enhancing the precision and effectiveness of MRT. However, uncertainties in PBPK model outputs, as a central component of digital twins, may affect the robustness of both therapeutic outcome prediction and administered activity optimization, necessitating systematic approaches for their evaluation and mitigation.

Sensitivity analysis (SA) offers a systematic framework to estimate uncertainties in PBPK modeling by evaluating how variations in model input parameters, both individually and through their interactions, affect model output. By quantifying the magnitude and impact of these uncertainties, SA enables a rigorous assessment of model reliability and predictive performance. Regulatory authorities, including the United States Food and Drug Administration (FDA)^12^ and the European Medicines Agency (EMA),^13^ emphasize the use of SA as a critical component of PBPK model evaluation to demonstrate model robustness and assess the clinical relevance of parameter uncertainty.

The SA approaches in clinical applications of PBPK modeling can be classified according to their objectives and the nature of parameter variability considered, into inter‐ and intra‐patient SA. Inter‐patient SA examines the variability of patient‐related model parameters across a population and primarily addresses “between‐patient” variability. This approach is particularly useful during model development and can guide decisions on which parameters should be measured, fixed, or estimated individually. In contrast, intra‐patient SA quantifies how uncertainties in model parameters arising from limitations in parameter estimation propagate to model outputs within a single patient. This makes intra‐patient SA especially valuable for routine clinical PBPK applications, as it supports more reliable individual absorbed dose (AD) estimates for personalized therapy planning. Importantly, intra‐patient SA is not intended to replace inter‐patient SA. Rather, both analyses are complementary: inter‐patient SA identifies parameters with high relevance to population‐level variability in model outputs, whereas intra‐patient SA quantifies the contribution of these parameters to output uncertainty for an individual patient.

From another perspective, SA can be broadly divided into local and global methods. Local sensitivity analysis (LSA) typically applies a one‐at‐a‐time sampling, where the influence of small perturbations in individual parameters around nominal values on the model output is examined.^14^ This method is computationally efficient and straightforward, but it neglects parameter interactions and simultaneous variations, which can lead to misleading conclusions in complex models with significant parameter interactions.^15^, ^16^, ^17^ In contrast, global sensitivity analysis (GSA) explores the entire parameter space by simultaneously varying all parameters within plausible ranges.^18^ GSA quantifies both the individual contributions of parameters and their interactions, making it particularly suitable for high‐dimensional, nonlinear models such as PBPK models. Additionally, GSA streamlines the computational process without compromising accuracy and precision, thereby enhancing the computational efficiency of PBPK models.^19^

Classical variance‐based GSA, capable of handling nonlinear relationships between model inputs and outputs, typically relies on the assumption that model inputs are independent.^20^ However, whole‐body PBPK models often include correlated parameters, which can undermine the validity of standard GSA results. Consequently, accounting for such correlations is essential to obtain reliable insights into parameter influences. Several methods have been developed to extend variance‐based GSA to accommodate dependent inputs,^21^, ^22^, ^23^, ^24^, ^25^ including the approach proposed by Kucherenko specifically for PBPK models.^26^ For example, Liu et al. compared the Morris,^27^ Sobol,^28^ and Kucherenko methods using minimally constructed PBPK models for three drugs given orally, namely quinidine, alprazolam, and midazolam.^29^ They demonstrated that both Morris and Sobol, which assume independent inputs, misrepresented the influence of several key parameters, for instance, overestimating the role of the steady‐state volume of distribution on the area under the time‐activity curve (AUC) and underestimating the contribution of liver volume and specific metabolic processes. In contrast, the Kucherenko method, which incorporates parameter correlations, yielded different sets of influential parameters, highlighting the importance of considering parameter dependencies when applying GSA to PBPK models.

To our knowledge, intra‐patient GSA has rarely been applied to PBPK models in MRT. Such analyses are critical because they can identify the most influential parameters contributing to uncertainty in estimated ADs for a given patient, thereby providing a mechanistic basis to refine individual dosimetry and support patient‐specific therapy optimization. Moreover, uncertainty analysis of AD is clinically important, as emphasized by the European Association of Nuclear Medicine (EANM) in their guidance on good practice for clinical dosimetry reporting, in which reporting uncertainty in AD is highlighted as a basic requirement of scientific practice.^30^ Intra‐patient GSA can advance PBPK‐based digital twins from patient‐specific predictive models towards uncertainty‐aware models by quantifying uncertainty in model outputs, thereby enhancing the reliability and interpretability of patient‐specific predictions in personalized MRT. The present study focuses on intra‐patient GSA and specifically investigates the practical impact of incorporating parameter correlations into GSA, which has not been explored in PBPK models in MRT. To address this gap, Kucherenko's GSA is applied to a whole‐body PBPK model of PSMA‐targeted therapies with ^1^ ^7^ ^7^Lu, systematically evaluating for the first time how parameter dependencies affect model outputs.

## 2 MATERIALS AND METHODS

### 2.1 Patients and data acquisition

Data from ten patients with metastatic prostate cancer were included in this retrospective study. Written informed consent was obtained from all patients. The local ethics committee of the University Hospital Würzburg waived the requirement for further approval because the study involved existing routine hospital data and individual treatment attempts, provided that results are published in anonymized form (waiver number: Az 20211111 02).

All patients had undergone [^68^Ga]Ga‐PSMA‐I&T PET/CT on a Biograph mCT 64 system (Siemens Healthineers) (80 ± 20 min post‐injection (p. i.); 2.8 ± 0.8 nmol peptide amount; 117 ± 19 MBq), followed by a first cycle of [^177^Lu]Lu‐PSMA‐I&T therapy (87.6 ± 2.7 nmol; 6.2 ± 0.2 GBq) and two SPECT/CT scans on a Symbia Intevo Bold system (Siemens Healthineers; dual‐head gamma camera with 9.5‐mm NaI(Tl) crystals) acquired at 1 and 4 days p. i. In addition, 3–4 whole‐body activity measurements were acquired during hospitalization using a dedicated ceiling‐mounted probe‐based whole‐body counting system.^31^

The PET/CT system had been harmonized through the EARL ^68^Ga accreditation, while a local clinical acquisition protocol was used (2 min/bed, 3D OSEM reconstruction without time‐of‐flight and with Siemens HD·PET point‐spread‐function modeling; 4.1 × 4.1 × 5.0 mm voxel size; CT‐based attenuation correction; 3 iterations, 21 subsets; 2‐mm Gaussian post‐filter). PET/CT imaging was performed as a whole‐body acquisition, providing axial coverage from the level of the eyes to the mid‐thigh. The SPECT/CT system had been quantitatively calibrated according to Tran‐Gia et al.^32^ Depending on the disease distribution, SPECT/CT acquisition was performed either using a single bed position centered on the abdominal region, providing axial coverage of the liver, spleen, kidneys, and tumors, or using extended acquisitions covering multiple bed positions from the neck to the upper thigh. Additional SPECT acquisition and reconstruction parameters were as follows: medium‐energy low‐penetration (MELP) collimators, 180° configuration, automatic contouring, continuous mode, 60 views/head (30 s/view), 256 × 256 matrix, and three energy windows (20% around the 208‐keV photopeak with two adjacent 10% scatter windows); xSPECT Quant reconstruction with 2.0‐mm isotropic voxels, CT‐based attenuation correction, triple‐energy‐window scatter correction, resolution modeling, 48 iterations and 1 subset, and no post‐filtering. For both modalities, low‐dose CT scans were acquired for attenuation correction and anatomical localization.

For the [^68^Ga]Ga‐PSMA‐I&T PET/CT and both [^177^Lu]Lu‐PSMA‐I&T SPECT/CT datasets, kidneys, liver, and spleen were initially segmented on CT using TotalSegmentator^33^ (version 2.0) and subsequently refined in 3D Slicer (version 5.6.1) to obtain accurate organ volumes. Segmented volumes were visually inspected in all three orthogonal planes to ensure anatomical plausibility. Each kidney was segmented as a single anatomical structure, consistent with the PBPK kidney subcompartments representing kinetic processes rather than anatomically distinct cortical or medullary regions. In addition, two PSMA‐positive lesions per patient were manually segmented in 3D Slicer; only lesions within the field of view of both PET/CT and post‐therapy SPECT/CT images that exhibited sufficiently high standardized uptake value (SUV), clear delineation, and no relevant overlap with adjacent PSMA‐avid tissues were included, thereby enabling reliable activity quantification. Isotropic expansions of all volumes of interest (VOIs) were generated to improve inclusion of spill‐out counts for PET and SPECT quantification. The degree of expansion was individually adjusted based on organ/lesion size, surrounding anatomy, and uptake distribution, with the aim of including activity originating from the structure itself while minimizing contamination from adjacent high‐uptake regions. The VOI strategy was informed by prior experience with NEMA image‐quality phantom measurements.^32^ A representative example of organ and lesion VOIs overlaid on PET/CT and post‐therapy SPECT/CT images is provided in Figure 1. Lastly, total‐body PSMA‐avid tumor volume (PSMA‐TV) was determined using a semiautomatic SUV‐threshold algorithm (fixed SUV = 3) implemented with the Beth Israel plug‐in in FIJI (ImageJ). All segmentations, including PSMA‐TV, were reviewed by a board‐certified nuclear medicine physician with expertise in PSMA‐targeting radiopharmaceuticals.

**FIGURE 1:**
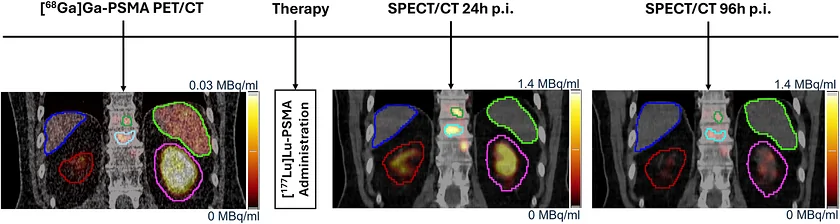
Representative example of lesion and organ segmentation in one patient. Coronal views of pre‐therapeutic [^6^ ^8^Ga]Ga‐PSMA‐I&T PET/CT and post‐therapy [^1^ ^7^ ^7^Lu]Lu‐PSMA‐I&T SPECT/CT acquired at 1 and 4 days post‐injection are shown with overlaid VOIs. VOIs included two selected tumor lesions, kidneys, liver, and spleen.

### 2.2 Model structure

In this study, the baseline model was a published whole‐body PBPK model in MATLAB Simbiology, originally developed for ^177^Lu‐labelled therapies targeting PSMA.^34^ A schematic overview of the PBPK model structure and the associated physiological parameters are presented in Figure S1 and Table S1 of the supplementary material, respectively. The model incorporated all major physiological and physical mechanisms, including blood flow, extravasation, specific binding, internalization, degradation, physical decay, and clearance from organs. Labelled and unlabeled peptides were modelled independently, assuming identical physiological parameters. Their interaction was represented through competitive binding to available receptor sites and the physical decay of the radiolabeled compound. The albumin binding property of the PSMA‐I&T radioligand was also considered in the model.

In the updated PBPK model used in this study, the representation of degradative intracellular transport via the endosome–lysosome pathway was refined for all organs and tumors expressing PSMA, along with an improved kidney submodel. The model also includes both [^6^ ^8^Ga]Ga‐PSMA‐I&T and [^1^ ^7^ ^7^Lu]Lu‐PSMA‐I&T biokinetics, capturing the sequential clinical workflow in which patients first undergo diagnostic [^6^ ^8^Ga]Ga‐PSMA PET/CT imaging followed by [^1^ ^7^ ^7^Lu]Lu‐PSMA therapy after a delay of several weeks. Two tumors and a tumor rest, defined as PSMA‐TV minus these two tumors, were implemented in the model.

Model parameters that are well‐established and available from the literature or from direct patient measurements were fixed (Table S1). For the remaining parameters, inter‐patient SA previously conducted on the same model identified parameters with either negligible or substantial impact on model outputs.^35^, ^36^ Parameters with negligible influence were fixed, whereas highly sensitive parameters that could not be measured directly, including receptor densities, flow‐related parameters, and key kinetic rates, were estimated by simultaneously fitting the model to the [^68^Ga]Ga‐ and [^177^Lu]Lu‐PSMA‐I&T time‐activity data of each patient (Table 1).

**TABLE 1:** Investigated PBPK model parameters.

Parameters	Description	Unit
Fil‐Fraction	Filtered fraction of blood flow in kidneys	unity
RD‐KID	PSMA receptor density in kidneys	nmol/L
RD‐LIV	PSMA receptor density in liver	nmol/L
RD‐SPL	PSMA receptor density in spleen	nmol/L
RD‐GLA	PSMA receptor density in salivary glands	nmol/L
RD‐TU1	PSMA receptor density in Tumor 1	nmol/L
RD‐TU2	PSMA receptor density in Tumor 2	nmol/L
CF‐RD‐RT	PSMA receptor density scaling factor in tumor rest	unity
FD‐TU1	Serum flow density in Tumor 1	L/min/kg
FD‐TU2	Serum flow density in Tumor 2	L/min/kg
CF‐FD‐RT	Serum flow density scaling factor in tumor rest	unity
K‐Deg	Degradation rate	1/min
Rel‐KID	Release rate in all organs	1/min
Rel‐TU1	Release rate in Tumor 1	1/min
Rel‐TU2	Release rate in Tumor 2	1/min

The quality of the time‐activity curves (TACs) estimated by the PBPK model was assessed using established goodness‐of‐fit criteria, including (i) visual inspection of agreement between measured time‐activity data and fitted TACs, (ii) quantitative assessment of the reliability of parameter estimates based on the coefficient of variation (CV < 50%, defined as the ratio of the standard error to the estimated parameter value), and (iii) evaluation of the parameter correlation matrix, requiring all off‐diagonal elements to have absolute values below 0.8 to confirm acceptable levels of parameter collinearity.^37^, ^38^ Upon confirmation of an acceptable goodness of fit, simulations were performed to compute the ADs of [^177^Lu]Lu‐PSMA‐I&T delivered to the kidneys, as the dose‐limiting organ, and to the tumors using the MIRD formalism^39^ as follows:

(1) $$AD_{k}\;\left(T\right)=A_{0}\times \;\tilde{a_{k}}\left(T\right)\times \;S\left(r_{k}\leftarrow r_{k}\right)$$

where $AD_{k}\left(T\right)$ represents the AD to region k, $A_{0}$ is the administered activity , $\tilde{a_{k}}\left(T\right)$ is the time‐integrated activity coefficient (TIAC) and $S(r_{k}\leftarrow r_{k})$ is the self‐dose factor for region k. In this study, AD calculations were performed at the organ and lesion level using self‐dose contributions only, which is a reasonable approximation for ^1^ ^7^ ^7^Lu due to its short beta‐particle range relative to organ/lesion dimensions.^40^ The ^177^Lu S‐values for each tumor and for the kidneys are summarized in Table S2. S‐values for tumors were derived using the sphere model implemented in OLINDA/EXM version 1.0, while kidney ADs were estimated using organ‐level, mass‐scaled S‐values within the MIRD framework.^41^

Kidney and tumor ADs were selected as the primary model outputs because they reflect principal determinants of treatment safety and efficacy in PSMA‐targeted MRT, while extension of the same framework to additional organs at risk would require sufficiently robust time‐activity data and validated organ‐specific model components. In this study, the intra‐individual uncertainty of the calculated ADs arises exclusively from uncertainty in the TAC fitting and the corresponding TIACs calculated using the PBPK model, whereas S‐values were assumed to be fixed to isolate the contribution of uncertainty associated with PBPK parameter estimation to the resulting AD estimates. This assumption aligns with previous findings by Zvereva et al.,^42^ who demonstrated that uncertainty in the AD estimation is predominantly driven by variability in TIACs, whereas the contribution of uncertainty associated with S‐values is comparatively minor.

### 2.3 Global sensitivity analysis

The Kucherenko method,^26^ as implemented in UQLab,^43^ was used to perform GSA. This method extends the widely used variance‐based Sobol GSA approach to scenarios with statistically dependent input parameters while retaining a framework well suited to nonlinear PBPK models.^26^, ^29^ In addition, it preserves the computation of main‐ and total‐effect sensitivity indices, enabling direct comparability with established Sobol and extended Fourier Amplitude Sensitivity Test (eFAST) indices. Alternative approaches for handling dependent input parameters have also been proposed, but may yield sensitivity measures that are less directly interpretable in terms of variance contributions.^44^, ^45^ The Kucherenko method relies on the law of total variance and defines the sensitivity indices through a direct decomposition of the output variance.

Given a model output Y=M(X) with input variables X, which are partitioned into two complementary subsets $X_{i}$ and $X_{\omega }=X_{∼i}$, the total variance of Y is expressed as:

(2) $$Var\;\left[Y\right]=Var\left[E\left(Y|X_{i}\right)\right]+E\left[Var\left(Y|X_{i}\right)\right]$$

The main sensitivity index $S_{i}$ quantifies the proportion of variance explained solely by $X_{i},$ while the total effect index $S_{i}^{T}$ accounts for the variance contribution of $X_{i}$, including its interactions with $X_{\omega }$. In contrast to classical Sobol methods, Kucherenko's approach accommodates correlations among inputs by using conditional sampling. The estimators are implemented efficiently in UQLab, avoiding computationally expensive nested loops by reformulating the Monte Carlo estimators. The main‐effect index is estimated as:

(3) $$S_{i}=\frac{\frac{1}{N}\;\sum_{j=1}^{N}\left[M\left(x_{i,j},\;\;x_{\omega ,j}\right)\;.\;M\left(x_{i,j},\;{x^{\prime }}_{\omega ,\;j}\right)\right]\;-\;\frac{1}{N}\;\sum_{j=1}^{N}M{\left(x_{i,\;j},\;\;x_{\omega ,j}\right)}^{2}\;}{Var\;\left[Y\right]}$$

with the total variance given by:

(4) $$\mathrm{Va}r\left[Y\right]=\frac{1}{N}\;\sum_{l=1}^{N}M{\left(x_{l}\right)}^{2}-{\left(\frac{1}{N}\sum_{l=1}^{N}M\left(x_{l}\right)\right)}^{2}$$

where ${x^{\prime }}_{\omega ,j}$ is sampled conditionally on $x_{i,j}$ and N is the number of Monte Carlo samples. The total‐effect index is estimated by:

(5) $$S_{i}^{T}=\frac{\frac{1}{N}\;\sum_{j=1}^{N}{\left[M\left(x_{i,\;j},\;x_{\omega .\;j}\right)-\;M\left({x^{\prime }}_{i,\;j},\;x_{\omega ,\;j}\right)\right]}^{2}}{2\;.\;Var\left[Y\right]}$$

The influence of uncertainty in the estimation of fifteen PBPK model parameters (listed in Table 1) on the model outputs, the ADs to the kidneys and tumors, was estimated using the Kucherenko GSA method. All parameters were modelled as lognormally distributed to ensure positivity. The mean of each distribution was defined based on the estimated parameter value obtained from the fitting process, and the standard deviation (SD) was derived from the corresponding standard error of the estimate. To account for statistical dependencies among the input parameters, a Gaussian copula was employed,^46^ with its correlation matrix defined by the pairwise parameter correlations estimated during the model fitting process.

In one approach of the analysis, parameter correlations were ignored by setting the Gaussian copula to the identity matrix (Figure 2; Approach 1). In a second approach, correlations were incorporated to reflect realistic parameter distributions and capture indirect effects arising from interdependencies (Figure 2; Approach 2). For both scenarios, pharmacokinetic simulations were repeated by sampling the parameters from their respective distributions according to a predefined sample size, and ADs to the kidneys and tumors were estimated. Subsequently, the main $\left(S_{i}\right)$ and total effect ($S_{i}^{T}$) sensitivity indices were calculated, quantifying the influence of each input parameter on the AD estimation.

**FIGURE 2:**
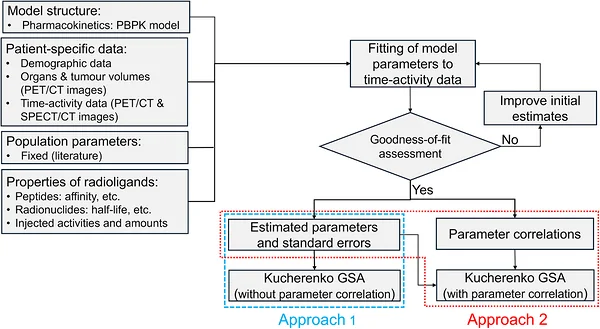
Overview of the PBPK model, parameter estimation process, and global sensitivity analysis (GSA) workflow. The PBPK model integrates physiological population data, patient‐specific data, and radioligand properties. Patient‐specific parameters that cannot be measured directly are estimated by fitting the model to imaging‐derived time–activity data, with iterative refinement of initial parameter values and evaluation of goodness‐of‐fit criteria. The resulting parameter estimates, together with their standard errors and correlation matrix, serve as inputs for intra‐patient GSA. Two approaches are shown: GSA without incorporation of parameter correlations (Approach 1), assuming independent parameters, and GSA with parameter correlations (Approach 2), accounting for statistical dependencies between parameters.

To determine an appropriate number of sampled input parameter sets required to achieve convergence in the GSA, a sequential approach was employed.^47^ In this approach, the number of samples is increased stepwise, and the stability of the sensitivity indices is visually and quantitatively examined by evaluating the convergence of sensitivity indices across all model outputs. In this study, the quantitative assessment proposed by Vanrolleghem et al. was performed.^48^ For each model output j, the ($SS_{ij}^{T}$) as the sum of the sensitivity indices of all parameters normalized to the number of parameters ($N_{p}$) was computed. The variability of $SS_{ij}^{T}$ ($V_{j})$ for the model output j between two consecutive sampling iterations K−1 and K was defined as:

(6) $$V_{j}=\left[\frac{{\left(\sum_{i=1}^{N_{p}}S_{i,j}^{T}\right)}_{K-1}-{\left(\sum_{i=1}^{N_{p}}S_{i,j}^{T}\right)}_{K}}{N_{p}}\right]\;$$

Convergence was considered achieved at sampling iteration K−1 if the variability $V_{j}$ remained within ± 1%^36^ for all model outputs when comparing $SS_{ij}^{T}$ at sampling iterations K−1 and K.

Intra‐patient uncertainty in AD estimates was defined as the SD of the AD distribution obtained from GSA for kidneys and tumors on a per‐patient basis. Additional uncertainty metrics were derived, including the CV and the width of the 95% confidence interval (CI), defined as the difference between the lower and upper bounds of the interval expected to contain the estimated AD with a probability of 95%. The calculated ADs and their corresponding uncertainties (SD) were compared between the two approaches across the cohort using paired Wilcoxon signed‐rank tests, considering p‐values below 0.05 as statistically significant. In addition, agreement between the two approaches was evaluated using Lin's concordance correlation coefficient (LCCC), which quantifies the degree of concordance between two measures.^49^ LCCC values range from −1 to +1, with higher values indicating stronger concordance. According to established criteria, LCCC>0.99 denotes almost perfect agreement, 0.95<LCCC<0.99 indicates substantial agreement, 0.90<LCCC<0.95 reflects moderate agreement, and LCCC<0.90 is considered poor concordance.

## 3 RESULTS

The goodness‐of‐fit assessment indicated overall acceptable agreement between measured time–activity data and the fitted TACs for the investigated organs/tumors across all patients. The biokinetic data and the corresponding fitted TACs for [^68^Ga]Ga‐PSMA‐I&T and [^1^ ^7^ ^7^Lu]Lu‐PSMA‐I&T in kidneys, liver, spleen, Tumor 1, Tumor 2, and whole body of a representative patient are presented in Figures S2–S3 of the supplementary material. The CV was below 50% for all fitted parameters, except for one patient, for whom the tumor receptor density and serum flow density exhibited CVs of 0.9 and 0.7, respectively. The off‐diagonal elements of the parameter correlation matrix ranged between –0.58 and 0.68 in all individual fits, indicating no excessive parameter collinearity. Figure 3 displays the median and interquartile range (IQR) of parameter correlation matrices across 10 patients, illustrating the typical parameter relationships and the variability in these correlations within the patient cohort.

**FIGURE 3:**
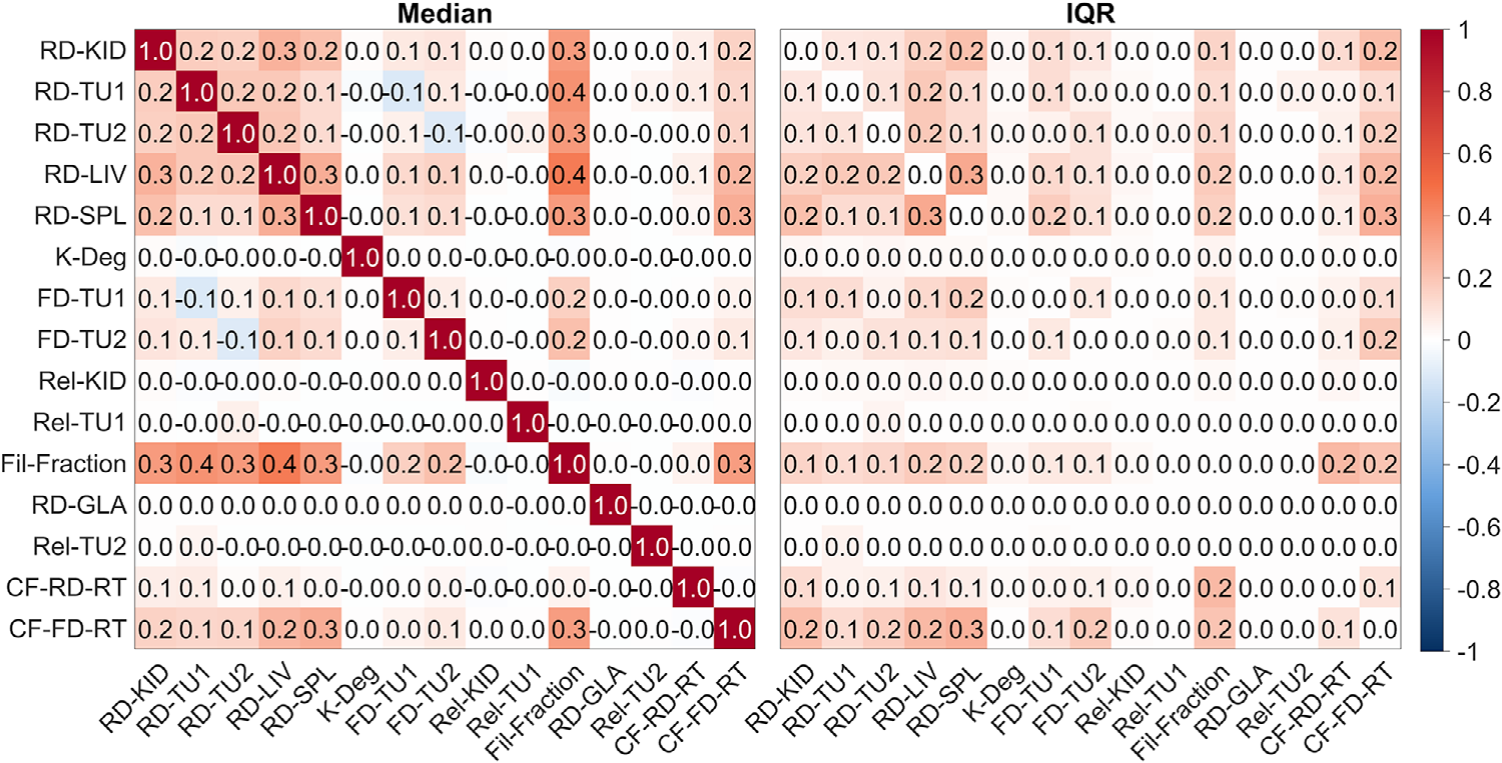
Heatmaps of median and interquartile range (IQR) of parameter correlation matrices derived from fitting the PBPK model parameters (Table 1) across 10 patients. Values within each cell represent the median correlation coefficients between two parameters (left) and their variability among patients (right), reflecting population‐level central tendencies and heterogeneity in parameter interdependencies. Strong median correlations indicate consistent relationships across patients, whereas larger IQR values indicate greater variability in the strength of these correlations.

Sequential sampling of PBPK model input parameters with sample sizes of 64, 128, 512, 1024, 2048, and 4096 confirmed the convergence of sensitivity indices for both approaches of the GSA, performed with and without accounting for parameter correlations. The convergence evaluation demonstrated that a sample size of 2048 (requiring 50.8 h CPU time on an Intel Core i7‐13700K, 3.4 GHz, 16 cores) was sufficient to achieve stable sensitivity indices for all patients in the GSA without accounting for parameter correlation. In contrast, when parameter correlations were incorporated, convergence was achieved with 1024 samples (requiring 16.8 h CPU time on an Intel Core i7‐11700K, 2.5 GHz, 8 cores), corresponding to an approximate twofold reduction in required sample size and a substantial decrease in computational time. The influence of sampling size on the estimated $S_{i}$ and $S_{i}^{T}$ sensitivity indices, as well as on the calculated ADs and their SDs for a typical patient, is presented in Figures S4–S5 of the supplementary material.

Figure 4 illustrates the patient‐specific input parameter distributions used for the intra‐patient GSA in two patients, accounting for parameter correlations, generated via Latin Hypercube Sampling (sample size of 2048, log‐normal distributions), with each histogram depicting the frequency of sampled parameter values. Presenting both patients together allows for a direct visual comparison, highlighting that the parameter distributions and consequently the input space explored during intra‐patient GSA can vary between patients due to individual‐specific model parameters. The corresponding distributions without accounting for parameter correlations are provided in Figure S6.

**FIGURE 4:**
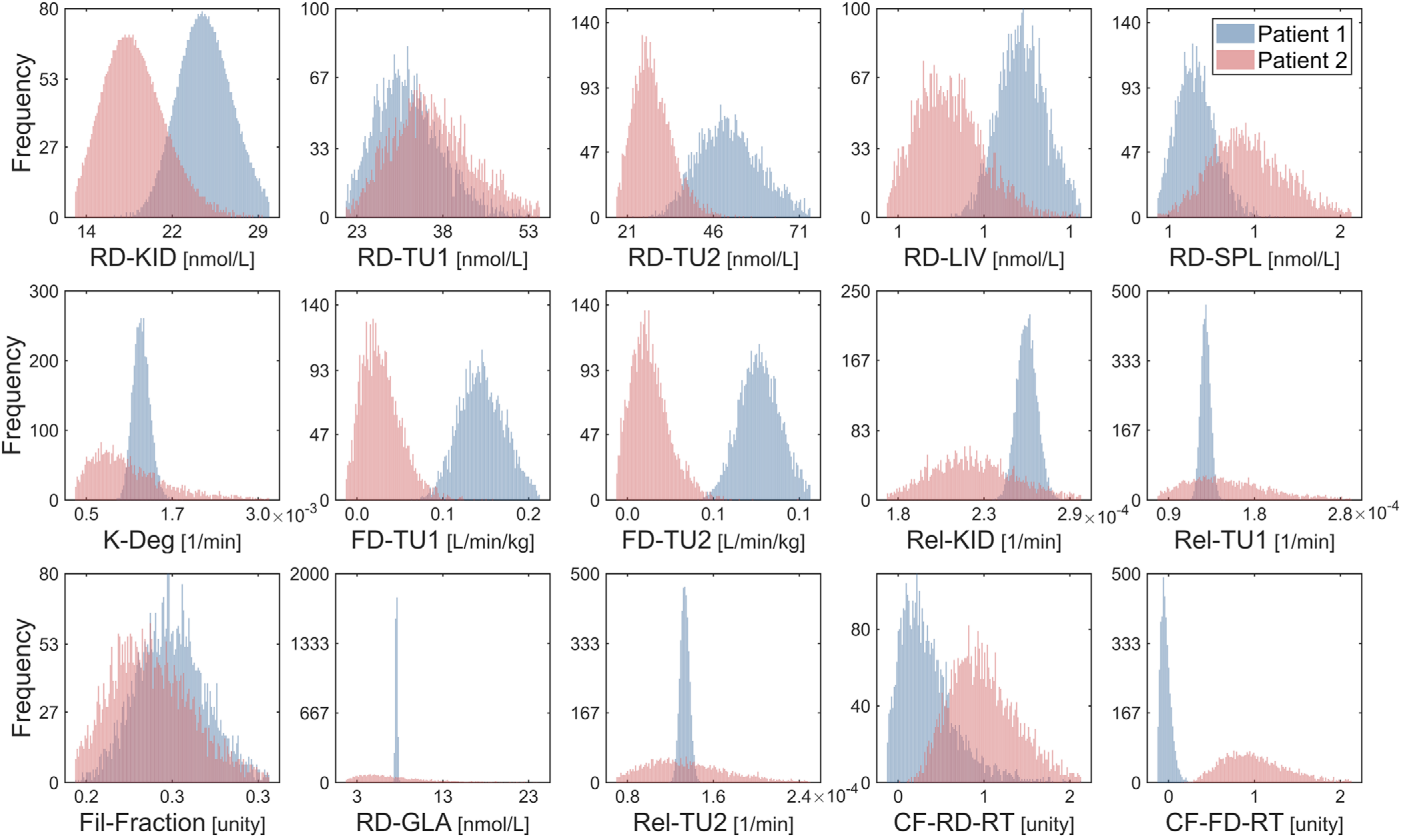
Histograms of the fifteen patient‐specific PBPK model parameter (Table 1) distributions used as input for the intra‐patient GSA in two randomly selected patients, accounting for parameter correlations. Each histogram shows the frequency of occurrence of sampled parameter values. Sampling was performed using the Latin Hypercube Sampling (LHS) method with log‐normal parameter distributions and a sample size of 2048. Displaying both patients in the same figure enables direct comparison of inter‐patient variability in the parameter distributions.

Figures 5 and 6 present a comparison of parameter‐specific $S_{i}^{T}$ and $S_{i}$ sensitivity indices, respectively, obtained from GSA performed with and without accounting for parameter correlations. Figure 5 shows that, in the presence of correlations, PSMA receptor density in the kidneys (RD‐KID) and the filtered fraction of renal blood flow (Fil‐Fraction) had the greatest influence on kidney ADs, followed by a moderate effect of tumor‐related parameters (RD‐TU2 and CF‐RD‐RT) in patients with larger tumor masses, with PSMA‐TV given as median [min–max]: 1.6 L [0.8–1.8 L]. The degradation rate (K‐Deg) had only a minor impact on kidney ADs, relevant for patients P2 and P9. For Tumors 1 and 2, the corresponding receptor densities (RD‐TU) were the dominant contributors to uncertainty in AD estimates. In patient P9, the release rates (Rel‐TU) of these tumors also had a notable effect. In the tumor rest, serum flow density and receptor density scaling factors (CF‐RD‐RT and CF‐FD‐RT), together with the receptor densities of Tumors 1 and 2, collectively contributed to AD uncertainty. When parameter correlations were excluded, the influence of RD‐KID on kidney ADs decreased slightly, whereas the contribution of Fil‐Fraction increased; nonetheless, both parameters remained the most influential. In tumors, the corresponding receptor densities continued to dominate uncertainty in AD estimates, with a slight increase in the effect of Fil‐Fraction and a reduced impact of release rates in patient P9.

**FIGURE 5:**
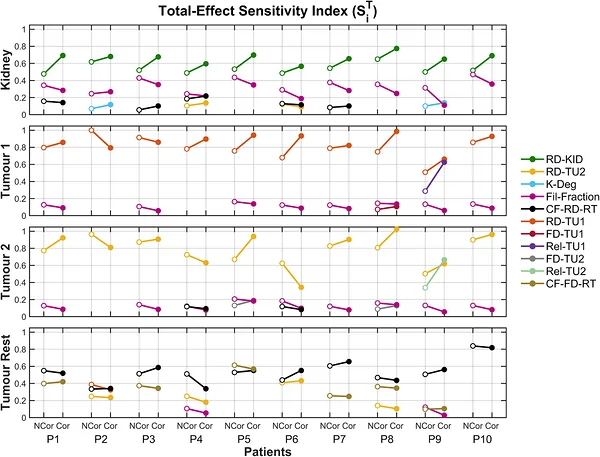
Paired comparison of parameter‐specific total‐effect sensitivity indices $\left(S_{i}^{T}\right)$ from GSA performed without parameter correlation (NCor) and with parameter correlation (Cor), with absorbed doses to the kidneys, Tumor 1, Tumor 2, and tumor rest as output variables in the sensitivity analysis. The indices quantify the total contribution of each parameter (Table 1) to the uncertainty of the model output, including both its direct effect and its interactions with other parameters. Only input parameters with $S_{i}^{T}$ values exceeding 0.1 are displayed for each patient. The results demonstrate that kidney receptor density (RD‐KID) and filtered fraction of renal blood flow (Fil‐Fraction) dominate kidney absorbed dose uncertainty, while tumor receptor densities (RD‐TU) are the primary drivers for tumor absorbed doses.

**FIGURE 6:**
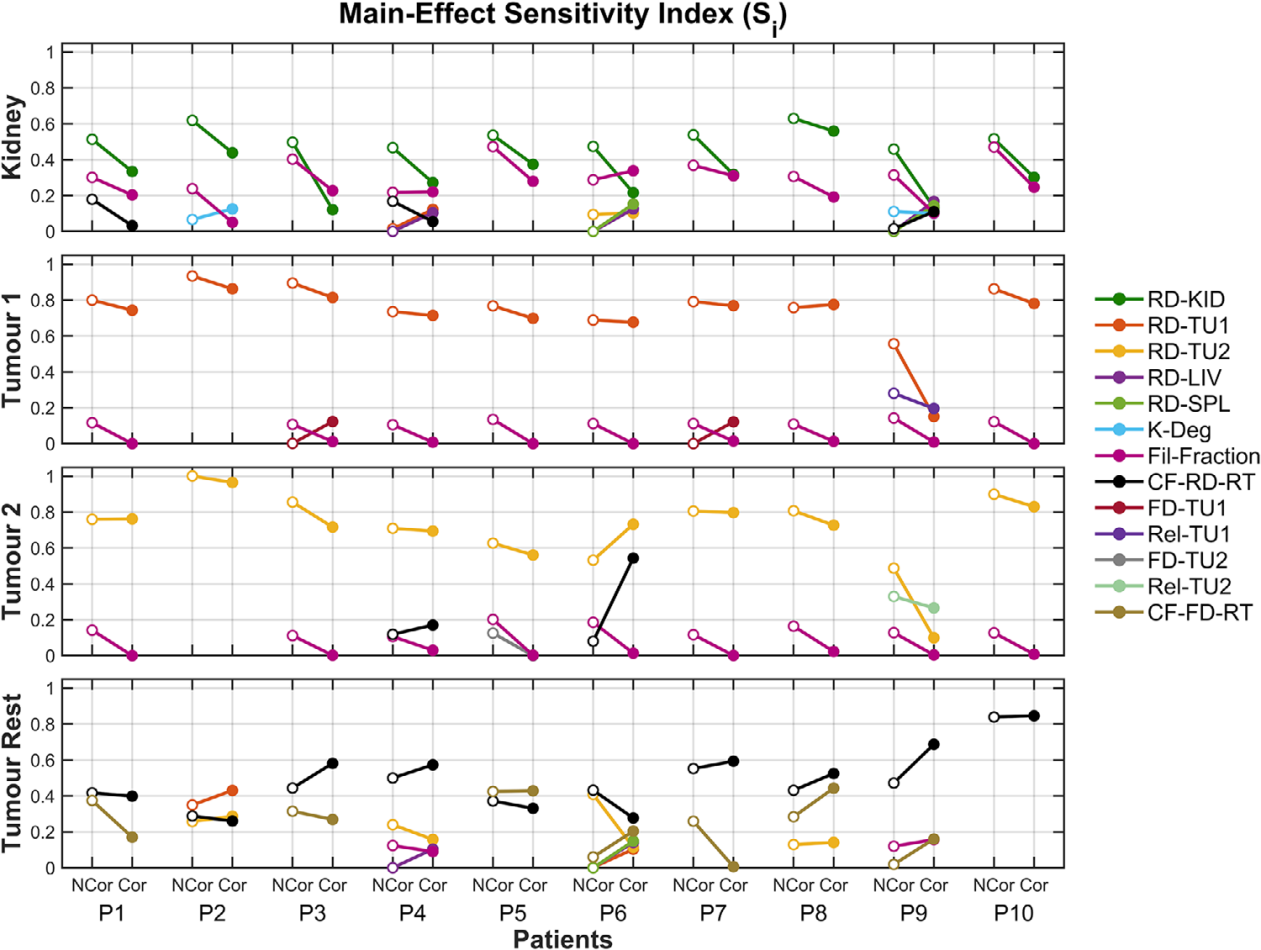
Paired comparison of parameter‐specific main‐effect sensitivity indices $\left(S_{i}\right)$ from GSA performed without parameter correlation (NCor) and with correlation (Cor), with absorbed doses to the kidneys, Tumor 1, Tumor 2, and tumor rest as output variables in the sensitivity analysis. The main‐effect indices quantify the direct contribution of each parameter (Table 1) to the uncertainty of the model output. Only input parameters with $S_{i}$ values exceeding 0.1 are displayed for each patient. Inclusion of parameter correlations alters the distribution of main effects but does not change the identity of the most influential parameters.

Figure 6 illustrates that a similar pattern was observed for the $S_{i}$ sensitivity indices, where the most influential parameters for each output retained almost the same rank as in Figure 5. In the absence of parameter correlations, all parameters exhibited lower $S_{i}$ values than $S_{i}^{T}$. In contrast, when parameter correlations were included, minor to moderate effects of specific parameters became apparent that were absent in the total effect analysis. For instance, the contribution of receptor densities of the liver and spleen (RD‐LIV and RD‐SPL) to kidney AD was not reflected in the $S_{i}^{T}$ results.

Table 2 summarizes the calculated ADs to the kidneys and tumors across the patient cohort, along with the intra‐patient uncertainty of the ADs (SD), for GSA performed with and without accounting for parameter correlations (individual‐patient results for the kidneys and tumors are provided in Table S3, and liver and spleen ADs together with associated uncertainty metrics are summarized in Table S4). Incorporating parameter correlations resulted in kidney ADs of 3.0–7.2 Gy (SD: 0.4–0.9 Gy) and tumor ADs of 2.0–26.8 Gy (SD: 0.6–3.4 Gy). Omitting correlations significantly increased the uncertainty in ADs by a factor of 1.2 [1.0–1.8] for both kidneys and tumors, whereas mean ADs remained essentially unchanged. Paired‐sample Wilcoxon signed‐rank tests indicated no significant difference in kidney AD between the two GSA approaches (*p* = 0.064), whereas the SDs of kidney ADs were significantly lower when parameter correlations were considered. In contrast, for tumors, both ADs and their SDs were significantly different between the two GSA approaches (*p* < 0.001).

**TABLE 2:** ADs and associated uncertainty metrics (SD and CV and 95% CI width) for kidneys and tumors (Tu1 and Tu2) in the ten‐patient cohort, calculated using GSA with and without the incorporation of parameter correlations. Tumor ADs and associated uncertainty metrics were aggregated across all analyzed lesions from all patients, whereas kidney statistics were evaluated on a per‐patient basis, with both reported as cohort‐level summary statistics. Statistical analysis with paired Wilcoxon signed‐rank tests demonstrated no significant difference in kidney ADs between the two approaches (*p* = 0.064), whereas a significant reduction in the uncertainty of ADs in kidneys was observed after including parameter correlations (*p* = 0.002). In contrast, tumor ADs differed significantly between the two GSA approaches, both in their mean values and associated SDs (*p* < 0.001). Consistently, CVs and 95% CI widths were lower when correlations were incorporated.

	Parameter correlations	AD [Gy] median [min–max]	SD [Gy] median [min–max]	CV [%] median [min–max]	95% CI width median [min–max]
Kidneys	Yes	4.2 [3.0–7.2]	0.5^*^[0.4–0.9]	11 [9–21]	1.9 [1.4–3.4]
Kidneys	No	4.3 [3.0–7.3]	0.6 [0.5–1.2]	14 [11–28]	2.4 [2.0–4.5]
Tumors	Yes	10.1^**^ [2.0–26.8]	1.7^**^ [0.6–3.4]	16 [12–84]	6.5 [2.3–13.4]
Tumors	No	10.2 [2.0–27.0]	2.1 [0.8–4.3]	20 [16–85]	8.2 [3.1–16.7]

Abbreviations: AD, absorbed dose; CI, confidence interval; CV, coefficient of variation; GSA, global sensitivity analysis; SD, standard deviation.

*p < 0.01; **p < 0.001.

The CV was consistently higher for tumor ADs than for kidney ADs, regardless of whether correlations were considered. Over the ten patients, the CVs for tumor ADs were 20% [16%–85%] without correlations and 16% [12%–84%] with correlations, compared with 14% [11%–28%] and 11% [9%–21%] for kidney ADs, respectively. Similarly, 95% CI widths were reduced when correlations were included: 1.9 [1.4–3.4] compared with 2.4 [2.0–4.5] for kidney ADs and 6.5 [2.3–13.4] compared with 8.2 [3.1–16.7] for tumor ADs, with versus without correlation, respectively.

The agreement in the calculated SDs by GSA with and without incorporation of parameter correlations was poor, with $LCCC_{Kidney}=0.70$ and $\mathrm{LCC}C_{\mathrm{Tumor}}=0.86$, indicating low concordance in the estimated uncertainties between the two approaches. This finding is further supported by Bland–Altman analyses presented in Figure S7.

## 4 DISCUSSION

This study demonstrates the application of intra‐patient GSA to a PBPK model for MRT and extends PBPK‐based digital twin implementations by introducing an uncertainty‐aware modeling framework at the individual patient level. While existing PBPK model implementations predominantly focus on patient‐specific estimates or predictions of pharmacokinetics, the present framework enables quantitative characterization of uncertainty in model outputs through propagation of parametric uncertainty and incorporation of parameter dependencies. The results demonstrated that neglecting parameter dependencies in GSA leads to systematic overestimation of AD uncertainty in PSMA‐targeted therapy with ^1^ ^7^ ^7^Lu, particularly for tumors, while the identity of the most influential parameters remained largely unchanged. These findings indicate that considering parameter dependencies in future PBPK‐based dosimetry studies can improve the reliability of AD estimates.

Inter‐patient LSA and GSA approaches have been applied to whole‐body PBPK models developed for ^177^Lu‐labelled MRT targeting PSMA.^35^, ^36^, ^50^, ^51^ Abdollahi et al. performed an LSA to evaluate how various model parameters affect AUC, AD, and biologically effective dose (BED) in tumors and the organs at risk, namely the kidneys and salivary glands.^50^ Their analysis showed that ligand amount, tumor volume, receptor densities, association rate, internalization rate, and release rate are key determinants of AUC, AD, and BED in tumors and organs at risk. However, LSA may not adequately capture the effects of nonlinear interactions between parameters, which are often present in complex PBPK models.^52^ A practical application of inter‐patient GSA was demonstrated by Hardiansyah et al., who assessed the impact of organ receptor densities, blood flows, and release rates on AD variability in kidneys and tumors using the eFAST algorithm.^36^ Their analysis revealed that kidney blood flow and receptor density significantly affect renal ADs, while tumor receptor density was the most influential parameter for tumor ADs. However, intra‐patient GSA and the assessment of parameter‐correlation effects within GSA frameworks have rarely been applied to PBPK models in MRT.

From a computational perspective, incorporating correlations in the intra‐patient GSA offered a clear efficiency advantage. Convergence of sensitivity indices was achieved with approximately half the number of samples when correlations were included compared with the independent‐parameter approach (1024 vs. 2048), resulting in a substantial reduction in computational time of approximately threefold under the investigated hardware conditions. This improvement reflects a more efficient exploration of the parameter space when statistical dependencies are preserved, thereby avoiding redundant sampling of physiologically implausible parameter combinations. In the context of PBPK‐based dosimetry, this reduction in computational burden may facilitate both the practical application of patient‐specific uncertainty quantification of ADs and further methodological development toward potential clinical translation.

The intra‐patient GSA conducted in this study demonstrated that the absence of parameter correlations in the SA resulted in an overestimation of uncertainties in the ADs for kidneys and tumors (Table 2). This was further reflected by the agreement analysis, in which the LCCC values for the uncertainties in kidney and tumor ADs were 0.70 and 0.86, respectively, indicating poor concordance between the two approaches. Inclusion of correlations among parameters limited the range of sampled values, thereby preventing physiologically unlikely combinations and reducing the uncertainty of the outputs. This finding is in good agreement with De Carlo et al.,^53^ who applied a GSA on a tumor growth inhibition model, showing that incorporating correlations allowed a more accurate assessment of both direct and indirect parameter effects on model predictions. Another study has demonstrated that ignoring parameter correlations can significantly affect sensitivity indices: in variance‐based sensitivity analyses, uncertainty in model output may be over‐ or underestimated, while in derivative‐based analyses, the magnitude or even the sign of the sensitivity indices can be reversed, altering the predicted model behaviour.^54^

Particularly in our study, neglecting correlations led to higher uncertainty for tumor ADs than for kidney ADs, as reflected in both the CV and the 95% CI widths, likely due to greater inherent variability in tumor‐related parameters (Table 2). These findings are broadly consistent with a previous study that investigated the contributions of different sources of uncertainty in tumor ADs in MRT.^55^ In that study, the median relative uncertainty in tumor ADs associated with TIAC calculations was approximately 30%, representing the same source of uncertainty addressed in the present analysis. The observed CV in tumor ADs was 21% [16%–85%] without correlations and 17% [12%–84%] with correlations in this study.

Neglecting parameter correlation in GSA showed no statistically significant change in kidney AD but led to a statistically significant and systematic overestimation of the associated uncertainty (Table 2). In contrast, for tumors, incorporation of parameter correlations significantly affected both AD estimates and their associated uncertainties. However, the magnitude of these effects is moderate and not uniform across all patients (Table S3). Specifically, the incorporation of parameter correlations reduced the median uncertainty in AD estimates from 0.6 to 0.5 Gy for kidneys and from 2.1 to 1.7 Gy for tumors, while the impact on the 95% CI widths of the AD estimates was small. The observed reduction should therefore be interpreted chiefly as an improvement in the reliability and interpretability of patient‐specific AD estimates rather than as evidence that a small absolute difference in uncertainty would necessarily alter clinical patient management. Such refinement may be most relevant in situations where AD estimates approach organ‐at‐risk AD constraints or are used to compare potential treatment adaptations. For kidneys, as the dose‐limiting organ in MRT, commonly cited conservative limits are on the order of 23–28 Gy^40^, although these thresholds are largely extrapolated from external‐beam radiotherapy and are not firmly established for [^177^Lu]Lu‐PSMA radioligand therapy.^56^ The reduction in uncertainty of the estimated ADs observed in this study indicates that, within the range of parameter correlations observed in the analyzed cohort, the precision of AD estimates can be preserved even without explicitly considering correlations. However, application of the GSA method that incorporates correlations is highly recommended in cases where strong interdependencies between model parameters are present.^24^, ^29^, ^44^, ^57^

Regardless of whether correlations were considered in the GSA, RD‐KID and the Fil‐Fraction parameters had the strongest influence on kidney ADs in all patients. Tumor‐related parameters (RD‐TU2 and CF‐RD‐RT) had a moderate influence on kidney ADs in patients with larger tumor masses, likely due to the tumor sink effect, whereby high radioligand uptake by tumors reduces the availability of the radioligand to normal tissues, including the kidneys.^58^, ^59^ For Tumors 1 and 2, the corresponding RD‐TU were the main determinants of AD uncertainty. In the tumor rest, AD uncertainty arose from a combination of CF‐RD‐RT and CF‐FD‐RT, along with contributions from the receptor densities of Tumors 1 and 2. Since the receptor density in the tumor rest is calculated as an average of the receptor densities in Tumors 1 and 2 with an additional correction factor, contributions from these parameters to tumor‐rest ADs are expected. In prostate cancer, bone metastases are predominantly osteoblastic,^60^, ^61^ characterized by sinusoidal vessel morphology, a highly fenestrated endothelial cell layer, and reduced blood flow.^62^ As a result, tumor blood flow was not the dominant contributor to AD uncertainty in individual tumors. In contrast, because the tumor rest consists of a mixture of bone and other types of lesions, CF‐FD‐RT is an influential determinant of tumor‐rest AD uncertainty in certain patients. These findings are in line with recently published intra‐patient GSA of a PBPK model for [^177^Lu]Lu‐PSMA‐I&T MRT conducted with considering correlations between input parameters, which identified kidney receptor density, renal blood flow, and tumor receptor densities as key contributors to uncertainty in kidney and tumor ADs, respectively.^63^ The difference in the kidney‐related parameter, renal blood flow in the previous study versus filtered fraction of renal blood flow in this study, likely reflects differences in the kidney model structure. In addition, the previous study identified tumor release rate as a secondary contributor to tumor AD uncertainty across the analyzed cohort, whereas in the present study, the corresponding tumor release parameters (Rel‐TU1 and Rel‐TU2) contributed substantially to tumor AD uncertainty in only a single patient (P9). These differences, together with the notable inter‐patient variability observed in both $S_{i}^{T}$ and $S_{i}$ in Figures 5 and 6, indicate that the contribution of individual parameters to uncertainty in kidney and tumor ADs, as well as their interactions with other parameters, can vary substantially between patients.

In the absence of parameter correlations, all parameters exhibited slightly lower $S_{i}$ values than $S_{i}^{T}$, as expected from the relationship $S_{i}^{T}$≥ $S_{i}$, given that $S_{i}^{T}$ includes both the main effect and all parameter interactions (Figures 5 and 6). The correspondence between $S_{i}$ and $S_{i}^{T}$ indicates that no substantial higher‐order effects were observed in the model. Once correlations were introduced, however, the interpretation of main and total effects became more challenging. $S_{i}$​ no longer reflected the purely independent influence of each parameter, as part of their contribution could be shared with correlated parameters. This could explain the apparent direct contribution of RD‐LIV and RD‐SPL to kidney ADs in some patients when parameter correlations are included in the GSA (Figure 6), which arises from their correlations with the most influential kidney parameters, namely RD‐KID and Fil‐Fraction (Figure 3). Similarly, $S_{i}^{T}$ was not limited to the direct and interaction terms of a single parameter but was also influenced by correlations, which could either enhance or diminish its magnitude relative to $S_{i}.$ For example, when kidney AD was considered as the output of the SA, $S_{i}^{T}$ decreased for RD‐LIV and RD‐SPL, so they were not among the parameters with a total effect greater than 0.10 (Figure 5), while it increased for RD‐KID compared to $S_{i,}$ indicating that the inequality $S_{i}^{T}$≥ $S_{i}$ is not guaranteed. This demonstrates that, although GSA with correlated inputs represents more realistic conditions, caution is required when interpreting the indices, as their meaning is less straightforward than in the uncorrelated setting.Model parameters with CVs exceeding 50%, thereby not fulfilling the predefined goodness‐of‐fit criterion (CV < 50%), indicate limited identifiability and result in broader input parameter distributions in the intra‐patient GSA, which may propagate into increased AD uncertainty and affect the robustness of the ranking of influential parameters. In the present cohort, this effect was observed in one patient (P2), where tumor‐related parameters exhibited high CVs, leading to markedly increased uncertainty in tumor AD estimates and only a limited reduction in uncertainty after accounting for parameter correlations (Table S3/). In addition, moderately elevated AD uncertainty was observed in tumor‐rest compartments of two patients (P5 and P10), despite all fitted parameters meeting the predefined goodness‐of‐fit criterion. No consistent patient‐level pattern could be identified to explain these cases, which may reflect more complex or heterogeneous tumor burden, although interpretation is limited by the small cohort size.

The present framework should not be regarded as a replacement for quantitative imaging or complementary pharmacokinetic sampling, but as a complementary approach that integrates these data and provides a mechanistic interpretation of the observed kinetics. While quantitative imaging provides observed time–activity data, it does not directly resolve latent parameters such as receptor density, filtration‐related transport, or their interdependencies. Additional blood or urine sampling may further constrain selected systemic or excretory processes and could therefore reduce uncertainty in future multimodal implementations of the framework.

Definitive prospective evidence demonstrating improved patient outcomes through dosimetry‐guided treatment adaptation in PSMA radioligand therapy remains limited. However, recent AD‐response studies in MRT increasingly support an association between tumor AD and treatment response, providing a biological basis for patient‐specific dosimetry.^64^, ^65^, ^66^, ^67^, ^68^ In this context, the present study provides a methodological framework for more reliable patient‐specific AD estimation. Uncertainty‐aware PBPK‐based digital twins may improve the interpretation of AD‐response relationships by explicitly accounting for the reliability of AD estimates through quantification of their uncertainty, which has been shown to influence the strength and interpretability of correlations between AD and therapeutic response.^69^ Furthermore, quantification of AD uncertainty may support adaptive treatment planning across therapy cycles by enabling assessment of whether observed inter‐cycle differences in AD reflect true physiological changes or fall within the expected uncertainty bounds. Collectively, these developments represent a conceptual step towards clinically actionable digital twins. However, prospective validation is required before such models can be implemented in routine clinical practice.

The findings of this study indicate that future PBPK‐based dosimetry workflows should incorporate uncertainty‐aware modeling by reporting uncertainty in model outputs, preserving and propagating parameter correlations, and using sensitivity rankings to prioritize parameter measurements that most effectively reduce AD uncertainty. At a methodological level, the presented framework is expected to be generalizable to other organs at risk and radioligand therapy models with dependent parameters. However, the magnitude of uncertainty in model outputs, the influence of parameter correlations on uncertainty reduction, and the identity of the dominant parameters are expected to be ligand‐, organ‐, and measurement‐protocol‐specific and depend additionally on the underlying model structure and the magnitude of parameter correlation. Notably, extension of the uncertainty analysis to additional organs at risk requires the availability of organ‐specific time‐activity data and validated PBPK submodels to support reliable quantitative interpretation. In addition, AD calculations in this study were performed using an organ‐ and lesion‐level MIRD‐based formalism with fixed S‐values, which does not account for spatial dose heterogeneity or cross‐organ dose contributions. While self‐dose is expected to dominate for ^1^ ^7^ ^7^Lu due to the short β‐particle range, cross‐dose may become relevant for other radionuclides or anatomical configurations. Although this approach provides a consistent basis for uncertainty propagation analysis, more advanced voxel‐based or Monte Carlo methods may improve accuracy in patient‐specific dosimetry, particularly in cases with heterogeneous activity distributions or significant cross‐dose effects.

Uncertainty in PBPK‐based digital twins may arise from several sources, including (i) experimental uncertainty related to image acquisition, reconstruction, segmentation, and activity quantification; (ii) structural/model uncertainty related to assumptions in the compartmental structure and governing equations; (iii) parametric uncertainty reflecting incomplete knowledge of patient‐specific parameter values; and (iv) numerical uncertainty related to solver tolerances and numerical implementation.^70^ The present study explicitly propagates parametric uncertainty and parameter correlations obtained from patient‐specific parameter fitting. Other sources of uncertainty were not varied separately; however, they are partially reflected in the parametric uncertainty. Accordingly, a comprehensive assessment of uncertainties in AD across the full dosimetry workflow remains challenging. In particular, this study omitted several sources of experimental uncertainty related to image reconstruction and quantification. Respiratory and patient motion were not explicitly modelled or propagated within the present GSA framework and may therefore contribute to additional unaccounted uncertainty in the AD estimates, particularly for tumors located near moving abdominal organs, where motion may affect the quantification of time‐activity data. The use of research‐oriented software tools for segmentation and image processing in this study is appropriate for methodological development; however, clinical implementation would require integration into validated and certified software environments. Furthermore, uncertainties associated with dose conversion factors (i.e., S‐values) were not considered. While this represents a limitation of the present work, prior studies indicate that TIAC‐related variability dominates AD uncertainty compared with uncertainties in S‐values.^42^ Therefore, the present results are expected to be robust with respect to the relative importance of model parameters and the impact of parameter correlations on AD uncertainties, although inclusion of S‐value uncertainty or other aforementioned experimental uncertainty sources could increase the absolute magnitude of the reported uncertainties and should be considered in future work.

The selection of lesions may influence the magnitude of the impact of parameter correlations on tumor AD uncertainty. This dependence on lesion selection is expected to be more pronounced for smaller lesions or spatially confluent lesions, due to reduced quantitative accuracy arising from partial volume effects and limited spatial resolution in ^177^Lu SPECT/CT. Another limitation of this study is the use of the Kucherenko approach to account for correlated input parameters in variance‐based GSA. Although it is currently one of the most suitable methods for PBPK models, ongoing methodological debate remains among GSA practitioners regarding the interpretation of results, not only for Kucherenko but also for other variance‐based GSA methods that assume dependent parameters.^44^, ^45^ Future methods that provide alternative ways to handle correlated parameters more effectively should also be explored in subsequent studies. In this study, post‐therapy imaging was available only for the first treatment cycle. Future studies analyzing patients undergoing multiple treatment cycles with available imaging data would enable evaluation of the inter‐cycle consistency of AD estimates, thereby providing further validation of the proposed approach, particularly with respect to the relative stability of kidney AD estimates and the evolution of tumor dosimetry across treatment cycles.

## 5 CONCLUSIONS

This study demonstrates that incorporating parameter correlations into intra‐patient GSA of a PBPK model for PSMA‐targeted therapies with ^177^Lu provides a more physiologically consistent propagation of parametric uncertainties to AD estimates. Neglecting correlations in GSA generally resulted in an overestimation of AD uncertainty, particularly for tumors, whereas incorporating correlations yielded lower uncertainty estimates for both kidney and tumor ADs and reduced the associated computational burden. Receptor densities in kidneys and tumors, together with the filtered fraction of renal blood flow, were consistently identified as the primary contributors to AD uncertainty, with additional patient‐specific differences emphasizing the need for individualized evaluation. These findings emphasize the importance of considering parameter correlations in PBPK‐based uncertainty and sensitivity analyses, although the interpretation of sensitivity indices becomes more complex under correlated conditions.

## CONFLICT OF INTEREST STATEMENT

Michael Lassmann has received institutional grants from Novartis and Pentixapharm. Johannes Tran‐Gia has received institutional grants from Novartis. Deni Hardiansyah and Gerhard Glatting serve as consultants for ITM Oncologics GmbH.

## Supporting information



Supporting Information

## References

[1] Sgouros G , Bodei L , McDevitt MR , Nedrow JR . Radiopharmaceutical therapy in cancer: clinical advances and challenges. Nat Rev Drug Discov. 2020;19(9):589‐608. doi:10.1038/s41573‐020‐0073‐9 10.1038/s41573-020-0073-9 PMC7390460 32728208

[2] Sharma S , Pandey MK . Radiometals in imaging and therapy: highlighting two decades of research. Pharmaceuticals. 2023;16(10):1460. doi:10.3390/ph16101460 10.3390/ph16101460 PMC10610335 37895931

[3] Sartor O , De Bono J , Chi KN , et al. [^177^Lu]Lu–PSMA‐617 for metastatic castration‐resistant prostate cancer. N Engl J Med. 2021;385(12):1091‐1103. doi:10.1056/NEJMoa2107322 10.1056/NEJMoa2107322 PMC8446332 34161051

[4] Strosberg J , El‐Haddad G , Wolin E , et al. Phase 3 trial of [^177^Lu]Lu‐dotatate for midgut neuroendocrine tumors. N Engl J Med. 2017;376(2):125‐135. doi:10.1056/NEJMoa1607427 10.1056/NEJMoa1607427 PMC5895095 28076709

[5] Parker C , Zhan L , Cislo P , et al. Effect of radium‐223 dichloride (Ra‐223) on hospitalisation: an analysis from the phase 3 randomised Alpharadin in Symptomatic Prostate Cancer Patients (ALSYMPCA) trial. Eur J Cancer. 2017;71:1‐6. doi:10.1016/j.ejca.2016.10.020 10.1016/j.ejca.2016.10.020 27930924

[6] Kletting P , Bunjes D , Reske SN , Glatting G . Improving anti‐CD45 antibody radioimmunotherapy using a physiologically based pharmacokinetic model. J Nucl Med. 2009;50(2):296‐302. doi:10.2967/jnumed.108.054189 10.2967/jnumed.108.054189 19164217

[7] Kletting P , Kull T , Bunjes D , et al. Radioimmunotherapy with Anti‐CD66 antibody: improving the biodistribution using a physiologically based pharmacokinetic model. J Nucl Med. 2010;51(3):484‐491. doi:10.2967/jnumed.109.067546 10.2967/jnumed.109.067546 20150257

[8] Rahmim A , Brosch‐Lenz J , Fele‐Paranj A , et al. Theranostic digital twins for personalized radiopharmaceutical therapies: reimagining theranostics via computational nuclear oncology. Front Oncol. 2022;12:1062592. doi:10.3389/fonc.2022.1062592 10.3389/fonc.2022.1062592 PMC9797662 36591527

[9] Brosch‐Lenz J , Uribe C , Rahmim A , Saboury B . Theranostic digital twins: an indispensable prerequisite for personalized cancer care. J Nucl Med. 2023;64(3):501. doi:10.2967/jnumed.122.264929 10.2967/jnumed.122.264929 PMC10071798 36863782

[10] Abdollahi H , Yousefirizi F , Shiri I , et al. Theranostic digital twins: concept, framework and roadmap towards personalized radiopharmaceutical therapies. Theranostics. 2024;14(9):3404‐3422. doi:10.7150/thno.93973 10.7150/thno.93973 PMC11209714 38948052

[11] Sinsoilliez N , Magnier B , Piron B , Bardiès M , Janaqi S , Boudousq V . Digital twins in nuclear medicine: a proposition of a modular pipeline for dosimetry protocol optimization in molecular radiotherapy. Comput Struct Biotechnol J. 2025;28:306‐311. doi:10.1016/j.csbj.2025.08.027 10.1016/j.csbj.2025.08.027 PMC12415080 40927645

[12] Center for Drug Evaluation and Research (CDER); U.S. Food and Drug Administration (FDA) . Physiologically Based Pharmacokinetic Analyses—Format and Content: Guidance for Industry . Report No.: FDA‐2016‐D‐3969. Published September 2018.

[13] Committee for Medicinal Product for Human Use (CHMP), European Medicines Agency (EMA ). Guideline on the Qualification and Reporting of Physiologically Based Pharmacokinetic (PBPK) Modelling and Simulation . . Report No.: EMA/CHMP/458101/2016. Published July 2016.

[14] Borgonovo E , Plischke E . Sensitivity analysis: a review of recent advances. Eur J Oper Res. 2016;248(3):869‐887. doi:10.1016/j.ejor.2015.06.032

[15] Campolongo F , Saltelli A . Sensitivity analysis of an environmental model: an application of different analysis methods. Reliab Eng Syst Saf. 1997;57(1):49‐69. doi:10.1016/S0951‐8320(97)00021‐5

[16] Saltelli A , Chan K , Scott EM . Handbook of Sensitivity Analysis. John Wiley & Sons publishers; 2000.

[17] Loizou G , Spendiff M , Barton HA , et al. Development of good modelling practice for physiologically based pharmacokinetic models for use in risk assessment: the first steps. Regul Toxicol Pharmacol. 2008;50(3):400‐411. doi:10.1016/j.yrtph.2008.01.011 10.1016/j.yrtph.2008.01.011 18331772

[18] Saltelli A , Ratto M , Andres T , et al. Global Sensitivity Analysis: The Primer. John Wiley & Sons; 2008.

[19] Hsieh NH , Reisfeld B , Bois FY , Chiu WA . Applying a global sensitivity analysis workflow to improve the computational efficiencies in physiologically‐based pharmacokinetic modeling. Front Pharmacol. 2018;9:588. doi:10.3389/fphar.2018.00588 10.3389/fphar.2018.00588 PMC6002508 29937730

[20] Prieur C , Tarantola S . Variance‐based sensitivity analysis: theory and estimation algorithms. In: Handbook of Uncertainty Quantification. Springer; 2017: 1217‐1239. doi:10.1007/978‐3‐319‐12385‐1_35

[21] Xu C , Gertner G . Extending a global sensitivity analysis technique to models with correlated parameters. Comput Stat Data Anal. 2007;51(12):5579‐5590. doi:10.1016/j.csda.2007.04.003

[22] Mara TA , Tarantola S , Annoni P . Non‐parametric methods for global sensitivity analysis of model output with dependent inputs. Environ Model Softw. 2015;72:173‐183. doi:10.1016/j.envsoft.2015.07.010

[23] Tarantola S , Mara TA . Variance‐based sensitivity indices of computer models with dependent inputs: the fourier amplitude sensitivity test. Int J Uncertain Quantif. 2017;7(6):511‐523. doi:10.1615/Int.J.UncertaintyQuantification.2017020291

[24] Do NC , Razavi S . Correlation effects? A major but often neglected component in sensitivity and uncertainty analysis. Water Resour Res. 2020;56(3):2019WR025436. doi:10.1029/2019WR025436

[25] Melillo N , Darwich AS . A latent variable approach to account for correlated inputs in global sensitivity analysis. J Pharmacokinet Pharmacodyn. 2021;48(5):671‐686. doi:10.1007/s10928‐021‐09764‐x 10.1007/s10928-021-09764-x PMC8405496 34032996

[26] Kucherenko S , Tarantola S , Annoni P . Estimation of global sensitivity indices for models with dependent variables. Comput Phys Commun. 2012;183(4):937‐946. doi:10.1016/j.cpc.2011.12.020

[27] Morris MD . Factorial sampling plans for preliminary computational experiments. Technometrics. 1991;33(2):161‐174. doi:10.2307/1269043

[28] Sobol′ IM . Global sensitivity indices for nonlinear mathematical models and their Monte Carlo estimates. Math Comput Simul. 2001;55(1):271‐280. doi:10.1016/S0378‐4754(00)00270‐6

[29] Liu D , Li L , Rostami‐Hodjegan A , Bois FY , Jamei M . Considerations and caveats when applying global sensitivity analysis methods to physiologically based pharmacokinetic models. AAPS J. 2020;22(5):93. doi:10.1208/s12248‐020‐00480‐x 10.1208/s12248-020-00480-x PMC7367914 32681207

[30] Lassmann M , Chiesa C , Flux G . EANM Dosimetry Committee guidance document: good practice of clinical dosimetry reporting. Eur J Nucl Med Mol Imaging. 2011;38(1):192‐200. doi:10.1007/s00259‐010‐1549‐3 10.1007/s00259-010-1549-3 20799035

[31] Lassmann M , Hänscheid H , Luster M , Reiners C . How to determine blood doses and remnant biokinetics for thyroid ablation therapy of differentiated thyroid cancer. Eur J Nucl Med Mol Imaging. 2004;31:259‐259.

[32] Tran‐Gia J , Denis‐Bacelar AM , Ferreira KM , et al. A multicentre and multi‐national evaluation of the accuracy of quantitative ^177^Lu SPECT/CT imaging performed within the MRTDosimetry project. EJNMMI Phys. 2021;8(1):55. doi:10.1186/s40658‐021‐00397‐0 10.1186/s40658-021-00397-0 PMC8302709 34297218

[33] Wasserthal J , Breit H‐C , Meyer MT , et al. TotalSegmentator: robust segmentation of 104 anatomic structures in CT images. Radiol Artif Intell. 2023;5(5):e230024. doi:10.1148/ryai.230024 10.1148/ryai.230024 PMC10546353 37795137

[34] Kletting P , Schuchardt C , Kulkarni HR , et al. Investigating the effect of ligand amount and injected therapeutic activity: a simulation study for ^177^Lu‐labeled PSMA‐targeting peptides. PLOS ONE. 2016;11(9):e0162303. doi:10.1371/journal.pone.0162303 10.1371/journal.pone.0162303 PMC5017739 27611841

[35] Hardiansyah D , Begum NJ , Kletting P , Mottaghy FM , Glatting G . Sensitivity analysis of a physiologically based pharmacokinetic model used for treatment planning in peptide receptor radionuclide therapy. Cancer Biother Radiopharm. 2016;31(6):217‐224. doi:10.1089/cbr.2016.2012 10.1089/cbr.2016.2012 27403777

[36] Hardiansyah D , Kletting P , Begum NJ , et al. Important pharmacokinetic parameters for individualization of ^177^Lu‐PSMA therapy: a global sensitivity analysis for a physiologically‐based pharmacokinetic model. Med Phys. 2021;48(2):556‐568. doi:10.1002/mp.14622 10.1002/mp.14622 33244792

[37] Kletting P , Schimmel S , Kestler HA , et al. Molecular radiotherapy: the NUKFIT software for calculating the time‐integrated activity coefficient. Med Phys. 2013;40(10):102504. doi:10.1118/1.4820367 10.1118/1.4820367 24089925

[38] Ivashchenko OV , O'Doherty J , Hardiansyah D , et al. Time‐Activity data fitting in molecular Radiotherapy: methodology and pitfalls. Phys Med. 2024;117:103192. doi:10.1016/j.ejmp.2023.103192 10.1016/j.ejmp.2023.103192 38052710

[39] Bolch WE , Eckerman KF , Sgouros G , Thomas SR . MIRD pamphlet No. 21: a generalized schema for radiopharmaceutical dosimetry—standardization of nomenclature. J Nucl Med. 2009;50(21):477‐484. doi:10.2967/jnumed.108.056036 10.2967/jnumed.108.056036 19258258

[40] Sjögreen Gleisner K , Chouin N , Gabina PM , et al. EANM dosimetry committee recommendations for dosimetry of ^177^Lu‐labelled somatostatin‐receptor‐ and PSMA‐targeting ligands. Eur J Nucl Med Mol Imaging. 2022;49(6):1778‐1809. doi:10.1007/s00259‐022‐05727‐7 10.1007/s00259-022-05727-7 PMC9015994 35284969

[41] Stabin MG , Sparks RB , Crowe E . OLINDA/EXM: the second‐generation personal computer software for internal dose assessment in nuclear medicine. J Nucl Med. 2005;46(6):1023‐1027. 15937315

[42] Zvereva A , Kamp F , Schlattl H , Zankl M , Parodi K . Impact of interpatient variability on organ dose estimates according to MIRD schema: uncertainty and variance‐based sensitivity analysis. Med Phys. 2018;45(7):3391‐3403. doi:10.1002/mp.12984 10.1002/mp.12984 29772064

[43] Marelli S , Sudret B . UQLab: a framework for uncertainty quantification in MATLAB. In: Vulnerability, Uncertainty, and Risk: Quantification, Mitigation, and Management. American Society of Civil Engineers; 2014: 2554‐2563. doi:10.1061/9780784413609.257

[44] Razavi S , Jakeman A , Saltelli A , et al. The Future of Sensitivity Analysis: an essential discipline for systems modeling and policy support. Environ Model Softw. 2021;137:104954. doi:10.1016/j.envsoft.2020.104954

[45] Pianosi F , Beven K , Freer J , et al. Sensitivity analysis of environmental models: a systematic review with practical workflow. Environ Model Softw. 2016;79:214‐232. doi:10.1016/j.envsoft.2016.02.008

[46] Nelsen RB . An Introduction to Copulas. Springer; 2006. doi:10.1007/0‐387‐28678‐0

[47] Sun X , Jakeman AJ , Croke BFW , Roberts SG , Jakeman JD . Assessing convergence in global sensitivity analysis: a review of methods for assessing and monitoring convergence. Socio‐Environ Syst Model. 2024;6:18678‐18678. doi:10.18174/sesmo.18678

[48] Vanrolleghem PA , Mannina G , Cosenza A , Neumann MB . Global sensitivity analysis for urban water quality modelling: terminology, convergence and comparison of different methods. J Hydrol. 2015;522:339‐352. doi:10.1016/j.jhydrol.2014.12.056

[49] Akoglu H . User's guide to correlation coefficients. Turk J Emerg Med. 2018;18(3):91‐93. doi:10.1016/j.tjem.2018.08.001 10.1016/j.tjem.2018.08.001 PMC6107969 30191186

[50] Abdollahi H , Fele‐Paranj A , Rahmim A . Model‐informed radiopharmaceutical therapy optimization: a study on the impact of pbpk model parameters on physical, biological, and statistical measures in ^177^Lu‐PSMA therapy. Cancers. 2024;16(18):3120. doi:10.3390/cancers16183120 10.3390/cancers16183120 PMC11430653 39335092

[51] Fele‐Paranj A , Saboury B , Uribe C , Rahmim A . Physiologically based radiopharmacokinetic (PBRPK) modeling to simulate and analyze radiopharmaceutical therapies: studies of non‐linearities, multi‐bolus injections, and albumin binding. EJNMMI Radiopharm Chem. 2024;9(1):6. doi:10.1186/s41181‐023‐00236‐w 10.1186/s41181-023-00236-w PMC10803696 38252191

[52] Saltelli A , Aleksankina K , Becker W , et al. Why so many published sensitivity analyses are false: a systematic review of sensitivity analysis practices. Environ Model Softw. 2019;114:29‐39. doi:10.1016/j.envsoft.2019.01.012

[53] De Carlo A , Tosca EM , Melillo N , Magni P . A two‐stages global sensitivity analysis by using the δ sensitivity index in presence of correlated inputs: application on a tumor growth inhibition model based on the dynamic energy budget theory. J Pharmacokinet Pharmacodyn. 2023;50(5):395‐409. doi:10.1007/s10928‐023‐09872‐w 10.1007/s10928-023-09872-w PMC10460734 37422844

[54] Kardoš J , Edeling W , Suleimenova D , Groen D , Schenk O . Sensitivity analysis of high‐dimensional models with correlated inputs. J Comput Sci. 2025;91:102681. doi:10.1016/j.jocs.2025.102681

[55] Finocchiaro D , Gear JI , Fioroni F , et al. Uncertainty analysis of tumour absorbed dose calculations in molecular radiotherapy. EJNMMI Phys. 2020;7(1):63. doi:10.1186/s40658‐020‐00328‐5 10.1186/s40658-020-00328-5 PMC7550507 33044651

[56] Kiess AP , O'Donoghue J , Uribe C , et al. How can radiopharmaceutical therapies reach their full potential? Improving dose reporting and phase i clinical trial design. J Clin Oncol. 2024;42(15):1734‐1737. doi:10.1200/JCO.23.01241 10.1200/JCO.23.01241 38484205

[57] Melillo N , Darwich AS , Magni P , Rostami‐Hodjegan A . Accounting for inter‐correlation between enzyme abundance: a simulation study to assess implications on global sensitivity analysis within physiologically‐based pharmacokinetics. J Pharmacokinet Pharmacodyn. 2019;46(2):137‐154. doi:10.1007/s10928‐019‐09627‐6 10.1007/s10928-019-09627-6 30905037

[58] Begum NJ , Thieme A , Eberhardt N , et al. The effect of total tumor volume on the biologically effective dose to tumor and kidneys for ^177^Lu‐labeled PSMA peptides. J Nucl Med. 2018;59(6):929‐933. doi:10.2967/jnumed.117.203505 10.2967/jnumed.117.203505 29419479

[59] Zamanian A , Rousseau É , Buteau F‐A , et al. The tumour sink effect on 68Ga‐PSMA‐PET/CT in metastatic castration‐resistant prostate cancer and its implications for PSMA‐RPT: a sub‐analysis of the 3TMPO study. Cancer Imaging. 2025;25:91. doi:10.1186/s40644‐025‐00910‐z 10.1186/s40644-025-00910-z PMC12261768 40665456

[60] Charhon SA , Chapuy MC , Delvin EE , Valentin‐Opran A , Edouard CM , Meunier PJ . Histomorphometric analysis of sclerotic bone metastases from prostatic carcinoma special reference to osteomalacia. Cancer. 1983;51(5):918‐924. doi:10.1002/1097‐0142(19830301)51:5%3C918::aid‐cncr2820510526%3E3.0.co;2‐j 10.1002/1097-0142(19830301)51:5<918::aid-cncr2820510526>3.0.co;2-j 6681595

[61] Roudier MP , Morrissey C , True LD , Higano CS , Vessella RL , Ott SM . Histopathological assessment of prostate cancer bone osteoblastic metastases. J Urol. 2008;180(3):1154‐1160. doi:10.1016/j.juro.2008.04.140 10.1016/j.juro.2008.04.140 PMC2992811 18639279

[62] Raymaekers K , Stegen S , van Gastel N , Carmeliet G . The vasculature: a vessel for bone metastasis. BoneKEy Rep. 2015;4:742. doi:10.1038/bonekey.2015.111 10.1038/bonekey.2015.111 PMC4859769 27217954

[63] Maudah AHS , Hardiansyah D , Yousefzadeh‐Nowshahr E , et al. Global sensitivity analysis of whole‐body physiologically based pharmacokinetic model parameters in [^177^Lu]Lu‐PSMA‐I&T radioligand therapy: key determinants of individual kidney and tumor absorbed dose uncertainty and implications for digital twin development. IEEE Trans Radiat Plasma Med Sci. 2026:1‐1. doi:10.1109/TRPMS.2026.3690503

[64] Strigari L , Konijnenberg M , Chiesa C , et al. The evidence base for the use of internal dosimetry in the clinical practice of molecular radiotherapy. Eur J Nucl Med Mol Imaging. 2014;41(10):1976‐1988. doi:10.1007/s00259‐014‐2824‐5 10.1007/s00259-014-2824-5 24915892

[65] Grkovski M , Krebs SS , O'Donoghue JA , et al. Lesion absorbed dose–response relationship in patients with metastatic castration‐resistant prostate cancer undergoing [^177^Lu]Lu‐PSMA‐617 radiopharmaceutical therapy. J Nucl Med. 2025;66(10):1622‐1630. doi:10.2967/jnumed.125.270170 10.2967/jnumed.125.270170 PMC12487730 40774698

[66] Hebert K , Santoro L , Monnier M , et al. Absorbed dose‐response relationship in patients with gastroenteropancreatic neuroendocrine tumors treated with [^177^Lu]Lu‐DOTATATE: one step closer to personalized medicine. J Nucl Med Off Publ Soc Nucl Med. 2024;65(6):923‐930. doi:10.2967/jnumed.123.267023 10.2967/jnumed.123.267023 PMC11149595 38637144

[67] Maccauro M , Cuomo M , Bauckneht M , et al. The LUTADOSE trial: tumour dosimetry after the first administration predicts progression free survival in gastro‐entero‐pancreatic neuroendocrine tumours (GEP NETs) patients treated with [^177^Lu]Lu‐DOTATATE. Eur J Nucl Med Mol Imaging. 2024;52(1):291‐304. doi:10.1007/s00259‐024‐06863‐y 10.1007/s00259-024-06863-y 39235614

[68] Warfvinge CF , Gustafsson J , Roth D , et al. Relationship between absorbed dose and response in neuroendocrine tumors treated with [^177^Lu]Lu‐DOTATATE. J Nucl Med. 2024;65(7):1070‐1075. doi:10.2967/jnumed.123.266991 10.2967/jnumed.123.266991 38724277

[69] Hu J , Seifert R , Karkampouna S , et al. Influence of dosimetry accuracy on the correlation with treatment outcome in a preliminary PSMA radiopharmaceutical therapy study. Eur J Nucl Med Mol Imaging. 2025;52(5):1649‐1657. doi:10.1007/s00259‐024‐07010‐3 10.1007/s00259-024-07010-3 PMC11928392 39704811

[70] Gargalo CL , de las Heras SC , Jones MN . Towards the Development of Digital Twins for the Bio‐manufacturing Industry. In: Herwig C , Pörtner R , Möller J , eds. Digital Twins: Tools and Concepts for Smart Biomanufacturing. Springer International Publishing; 2021:1‐34. doi:10.1007/10_2020_142 10.1007/10_2020_142 33349908

