# Supplementary material for: Impact of correlated PBPK parameters on intra‐patient global sensitivity analysis in molecular radiotherapy
Source: Med Phys. 2026 Nov 2;53(11):e70746. doi: 10.1002/mp.70746 (PMC13728525; doi:10.1002/mp.70746)
Supplement: Supplementary file 1 [file MP-53-0-s001.docx]

# Supplementary Material

# Impact of Correlated PBPK Parameters on Intra-Patient Global Sensitivity Analysis in Molecular Radiotherapy

Elham Yousefzadeh-Nowshahr^1,2^, Deni Hardiansyah^3^, Valentina Vasic^1^, Anna-Lena Theisen^4^, Johannes Tran-Gia^4^, Philipp E. Hartrampf^4^, Ambros J. Beer^2^, Michael Lassmann^4^, Gerhard Glatting^1,2^

^1^Medical Radiation Physics, Department of Nuclear Medicine, Ulm University, Ulm, Germany

^2^Department of Nuclear Medicine, University Hospital Ulm, Ulm, Germany

^3^Medical Physics and Biophysics, Physics Department, Faculty of Mathematics and Natural Sciences, Universitas Indonesia, Depok, Indonesia

^4^Department of Nuclear Medicine, University Hospital Würzburg, Würzburg, Germany

In this Supplement, the structural framework of the PBPK model and the corresponding physiological parameters are presented. The convergences of total- and main-effect sensitivity indices, absorbed doses, and their associated standard deviations, obtained through a sequential sampling approach, are reported. Moreover, inter-patient variability in absorbed doses and associated uncertainty metrics for kidneys and tumours is presented individually for all ten patients. Absorbed doses and uncertainty metrics for the liver and spleen are summarised at the cohort level across the 10 patients.

Figure S1. Structural layout of the PBPK model. a) Each organ is modelled as a rectangular compartment and linked through serum circulation. Renal clearance is considered the primary pathway for elimination. Since the peptide–albumin bound fraction is relatively small compared to the total peptide amount, and to keep the model structure simple, this compartment is connected only to the venous blood. b) Except for arteries, veins, the brain, and peptide-albumin binding, all organs are further subdivided into sub-compartments. A typical sub-compartment structure for PSMA-specific organs and tumours includes extravasation, specific binding, internalisation, sorting, degradation, recycling, release and physical decay.

Table S1: The associated physiological parameters

| Variable | | Value | Units | Ref |
| --- | --- | --- | --- | --- |
| $\boldsymbol{K}_{\boldsymbol{on}}$ | association rate | 0.046 | L/nmol/min | ^1,2^ |
| $\boldsymbol{K}_{\boldsymbol{d}}$ | dissociation constant | 1 | nmol/L | ^1,2^ |
| $\boldsymbol{K}_{\boldsymbol{off}}$ | dissociation rate | $K_{d}\times K_{on}$ | 1/min |  |
| $\boldsymbol{K}_{\boldsymbol{alb}}$ | peptide to albumin binding rate | 0.00047 | 1/min | ^3^ |
| $\boldsymbol{\lambda}_{\boldsymbol{phys}\mathbf{,}\boldsymbol{Ga}}$ | physical decay of ^68^Ga | 0.0102369 | 1/min | ^4^ |
| $\boldsymbol{\lambda}_{\boldsymbol{phys}\mathbf{,}\boldsymbol{Lu}}$ | physical decay of ^177^Lu | 0.0000724 | 1/min | ^5^ |
| Age | age | measured | years |  |
| BW | body weight | measured | kg |  |
| BH | body height | measured | cm |  |
| BSA | body surface area | 0.007184$\times$(BH^0.725^)$\times$(BW^0.425^) | $m^{2}$ | ^6^ |
| H | hematocrit | measured | unity |  |
| VTBS | total body serum volume | 2.8$\times$(1$-$H)$\times$BSA | L |  |
| F | total serum flow without tumour | VTBS$\times$1.23 | L/min | ^2,7^ |
| VART | arterial serum plus 1/2 serum content of heart | 0.06$\times$VTBS + 0.045$\times$VTBS | L | ^7^ |
| VVEN | venous serum plus 1/2 serum content of the heart | 0.18$\times$VTBS + 0.045$\times$VTBS | L | ^7^ |
| ρ | assumed density for all organs and tumours | 1 | mL/g |  |
| A-PET | injected activity in PET/CT | measured | MBq |  |
| A-Thera | injected activity in therapy | measured | MBq |  |
| PET-Cold | injected amount of unlabelled peptide in PET/CT | measured | nmol |  |
| PET-Hot | injected amount of labelled peptide in PET/CT | measured | nmol |  |
| Thera-cold | administered amount of unlabelled peptide in therapy | measured | nmol |  |
| Thera-hot | administered amount of labelled peptide in therapy | measured | nmol |  |
| inf-time | Therapy infusion time | 5 | min |  |
| t-lag | elapsed time between pre-therapeutic and therapy administration | measured | min |  |
| $\boldsymbol{k}_{\boldsymbol{int}}$ | internalisation rate in all organs and tumours | 0.001 | 1/min | ^8^ |
| $\boldsymbol{k}_{\boldsymbol{deg}}$ | degradation rate in all organs and tumours | fitted | 1/min |  |
| Tumours | | | | |
| MTU1 | volume of tumour 1 | measured on CT | L |  |
| MTU2 | volume of tumour 2 | measured on CT | L |  |
| MTuR | total volume of tumour-rest | measured on PET | L |  |
| CF-FD-RT | serum flow density scaling factor in residual tumour | fitted | unity |  |
| FD-TU1 | serum flow density in tumour 1 | fitted | L/min/kg |  |
| FD-TU2 | serum flow density in tumour 2 | fitted | L/min/kg |  |
| FD-TR | serum flow density in tumour-rest | CF-FD-RT$\times$ (FD-TU1 + FD-TU2) $\times$0.5 | L/min/kg |  |
| FTU1 | serum flow in the tumour 1 | FD-TU1$\times$MTU1 | L/min |  |
| FTU2 | serum flow in the tumour 2 | FD-TU2$\times$MTU2 | L/min |  |
| FTuR | serum flow in tumour-rest | FD-TR$\times$MTuR | L/min |  |
| kTU1 | permeability surface area product per unit mass for tumour 1 | 0.6 (maximum value from reference) | L/min/kg | ^9^ |
| kTU2 | permeability surface area product per unit mass for tumour 2 | 0.6 (maximum value from reference) | L/min/kg | ^9^ |
| kTuR | permeability surface area product per unit mass for tumour rest | 0.6 (maximum value from reference) | L/min/kg | ^9^ |
| CF-RD-TuR | Receptor density scaling factor in tumour-rest | fitted | unity |  |
| RD-TU1 | receptor density in tumour 1 | fitted | nmol/L |  |
| RD-TU2 | receptor density in tumour 2 | fitted | nmol/L |  |
| RD-TuR | receptor density in tumour | CF-RD-TuR $\times$  (RD-TU1 + RD-TU2) $\times$0.5 | nmol/L |  |
| Rel-TU1 | release rate in tumour 1 | fitted | 1/min |  |
| Rel-TU2 | release rate in tumour 2 | fitted | 1/min |  |
| VTui | interstitial volume in tumour | 0.38$\times$MTU | L | ^10^ |
| VTuv | vascular volume in tumour | 0.05$\times$ (1-H) $\times$MTu | L | ^9^ |
| VTBTi | interstitial volume in tumour-rest | 0.38$\times$MTuR | L | ^10^ |
| VTBTv | vascular volume in tumour-rest | 0.05$\times$ (1-H) $\times$MTuR | L | ^9^ |
| Rel-TuR | release rate in tumour-rest | (Rel-TU1 + Rel-TU2) / 2 | 1/min |  |
| Liver, spleen and kidneys | | | | |
| MLIV | total volume of liver | Measured on CT | L |  |
| MSPL | total volume of spleen | Measured on CT | L |  |
| MKID | total volume of kidneys | Measured on CT | L |  |
| MKID-Phan | total volume of kidneys in average human | 0.299 | L | ^11^ |
| RD-LIV | receptor density in liver | fitted | nmol/L |  |
| RD-SPL | receptor density in spleen | fitted | nmol/L |  |
| RD-KID | receptor density in kidneys | fitted | nmol/L |  |
| Rel-KID | release rate in kidneys | fitted | 1/min |  |
| VKID-cell | intercellular volume of kidneys | (MKID$-$VKIDi$-$VKIDv)$\times$2/3 | L | ^2^ |
| VKIDi | interstitial volume in kidneys | 0.15$\times$MKID | L | ^12^ |
| VKIDv | vascular volume in kidneys | 0.055$\times$MKID | L | ^12^ |
| VLIVi | interstitial volume in liver | 0.2$\times$MLIV | L | ^12^ |
| VLIVv | vascular volume in liver | 0.085$\times$MLIV | L | ^12^ |
| VSPLi | interstitial volume in spleen | 0.2$\times$MSPL | L | ^12^ |
| VSPLv | vascular volume in spleen | 0.12$\times$MSPL | L | ^12^ |
| fKC | kidney blood flow, age-independent factor | 4.1 | L/min/kg | ^13^ |
| fKID | serum flow density in kidney | fKC$-$(0.026$\times$Age) | L/min/kg | ^14^ |
| FKID | serum flow in kidneys | fKID$\times$MKID$\times$ (1$-$H) | L/min |  |
| Fil-fraction | filtered fraction of blood flow in kidneys | fitted | unity |  |
| GFR | glomerular filtration rate with ^51^Cr-EDTA | GFR = Fil-fraction$\times$FKID | L/min | ^15^ |
| KSC-PET | ratio of sieving coefficient diagnostic | 0.75 | unity | ^13^ |
| KSC_Thera |  | 0.66 | unity | ^16^ |
| Filtration | filtration rate in the kidney | GFR $\times$KSC | L/min |  |
| kLIV | permeability surface area product per unit mass for liver | 2 | L/min/kg | ^17^ |
| kSPL | permeability surface area product per unit mass for spleen | 2 (liver-like capillary structure) | L/min/kg |  |
| FLIV | serum flow in the liver | 0.065$\times$F | L/min | ^18^ |
| FSPL | serum flow in the spleen | 0.03$\times$F | L/min | ^18^ |
| Other Organs | | | | |
| MADI | total volume of adipose tissue | 13.465$\times$ (BW/71) | L | ^19^ |
| MBON | total volume of bone | (10.165$\times$ (BW/71))$-$MRBM | L | ^19^ |
| MBRA | total volume of brain | 1.45$\times$ (BW/71) | L | ^19^ |
| MGIT | total volume of gastrointestinal tract | (0.385+0.548+0.104+0.15) $\times$ (BW/71) | L | ^19^ |
| MHEA | total volume of heart | 0.341$\times$ (BW/71) | L | ^19^ |
| MLAC | total volume of lacrimal glands | 0.00068 | L | ^20^ |
| MLUN | total volume of the lung | 1$\times$ (BW/71) | L | ^19^ |
| MMUS | total volume of muscle | 30.078$\times$ (BW/71) | L | ^19^ |
| MPAR | total volume of parotid glands | 0.05 | L | ^21^ |
| MPRO | total volume of the prostate | 0.016$\times$ (BW/71) | L | ^19^ |
| MRBM | total volume of red bone marrow | 1.1$\times$ (BW/71) | L | ^19^ |
| MRES | total volume of remainder, i = all organs | BW$-\sum M_{i}$ | L | ^19^ |
| MSKI | total volume of skin | 3.408$\times$ (BW/71) | L | ^19^ |
| MSUB | total volume of submandibular | 0.025 | L | ^21^ |
| fLAC | serum flow density in lacrimal glands | 0.16 | L/min/kg | ^2^ |
| fPAR | serum flow density in parotid glands | 0.16 | L/min/kg | ^2^ |
| fSUB | serum flow density in submandibular glands | 0.16 | L/min/kg | ^2^ |
| fPRO | serum flow density in prostate | 0.18$\times$ (1-H) | L/min/kg | ^10^ |
| FADI | serum flow in adipose tissue | 0.05$\times$F | L/min | ^18^ |
| FBON | serum flow in bone | 0.05$\times$F | L/min | ^18^ |
| FBRA | serum flow in the brain | 0.12$\times$F | L/min | ^18^ |
| FGIT | serum flow in the gastrointestinal tract | 0.16$\times$F | L/min | ^18^ |
| FHEA | serum flow in the heart | 0.04$\times$F | L/min | ^18^ |
| FLAC | serum flow in lacrimal glands | fLAC$\times$MLAC | L/min |  |
| FMUS | serum flow in muscle | 0.17$\times$F | L/min | ^18^ |
| FPAR | serum flow in parotid glands | fPAR$\times$MPAR | L/min |  |
| FPRO | serum flow in the prostate | fPRO$\times$MPRO | L/min |  |
| FRBM | serum flow in red bone marrow | 0.03$\times$F | L/min | ^18^ |
| FRES | serum flow in the remainder, i = all organs | FRES = F $- \sum_{i} F_{i}$ | L/min |  |
| FSKI | serum flow in skin | 0.05$\times$F | L/min | ^18^ |
| FSUB | serum flow in the submandibular glands | fSUB$\times$MSUB | L/min |  |
| kMUS | permeability surface area product per unit mass for muscle | 0.02 | L/min/kg | ^17^ |
| kADI | permeability surface area product per unit mass for adipose tissue | 0.02 (assumed like muscle) | L/min/kg |  |
| kBON | permeability surface area product per unit mass for bone | 0.02 (assumed like muscle) | L/min/kg |  |
| kGIT | permeability surface area product per unit mass for the gastrointestinal tract | 0.02 (assumed like muscle) | L/min/kg |  |
| kHEA | permeability surface area product per unit mass for the heart | 0.02 (assumed like muscle) | L/min/kg |  |
| kLAC | permeability surface area product per unit mass for lacrimal glands | 0.4 (assumed like salivary glands) | L/min/kg |  |
| kLUN | permeability surface area product per unit mass for the lung | 2 | L/min/kg | ^17^ |
| kPAR | permeability surface area product per unit mass for parotid glands | 0.4 | L/min/kg | ^22^ |
| kPRO | permeability surface area product per unit mass for prostate | 0.1 | L/min/kg | ^10^ |
| kRBM | permeability surface area product per unit mass for red bone marrow | 2 (assumed like liver) | L/min/kg |  |
| kRES | permeability surface area product per unit mass for the remainder | 0.02 (assumed like muscle) | L/min/kg |  |
| kSKI | permeability surface area product per unit mass for skin | 0.02 (assumed like muscle) | L/min/kg |  |
| kSUB | permeability surface area product per unit mass for submandibular glands | 0.4 | L/min/kg | ^22^ |
| RD-GIT | receptor density in gastrointestinal tract | RD-PRO$\times$0.06 | nmol/L | ^23^ |
| RD-LAC | receptor density in lacrimal glands | assumed like parotid glands | nmol/L |  |
| RD-PAR | receptor density in parotid glands | fitted | nmol/L |  |
| RD-PRO | receptor density in prostate | 11 | nmol/L | ^2,24^ |
| RD-SUB | receptor density in submandibular glands | assumed like parotid glands | nmol/L |  |
| VADIv | vascular volume in adipose | 0.05$\times$VTBS | L | ^18^ |
| VADIi | interstitial volume in adipose | VADIv$\times$ (2289/148) | L | ^12^ |
| VBONv | vascular volume in Bone | (0.07$\times$VTBS)$-$VRBMv | L | ^18^ |
| VBONi | interstitial volume in bone | VBONv$\times$ (1891/224) | L | ^12^ |
| VBRAv | vascular volume in Brain | 0.012$\times$VTBS | L | ^18^ |
| VGITv | vascular volume in gastrointestinal tract | 0.076$\times$VTBS | L | ^18^ |
| VGITi | interstitial volume in gastrointestinal tract | VGITv$\times$ ((67.1+95.3+18)/(6.15+8.74+5.7)) | L | ^12^ |
| VHEAv | vascular volume in heart | 0.01$\times$VTBS | L | ^18^ |
| VHEAi | interstitial volume in heart | VHEAv$\times$ (48.8/13.1) | L | ^12^ |
| VLACi | interstitial volume in lacrimal glands | 0.23$\times$MLAC (assumed like salivary glands) | L |  |
| VLACv | vascular volume in lacrimal glands | 0.03$\times$ (1$-$H) $\times$MLAC (assumed like salivary glands) | L |  |
| VLUNi | interstitial volume in lung | VLUN$\times$(300/55) | L | ^12^ |
| VLUNv | vascular volume in lung | 0.105$\times$VTBS | L | ^18^ |
| VMUSi | interstitial volume in muscle | VMUSv$\times$ (3910/662) | L | ^12^ |
| VMUSv | vascular volume in muscle | 0.14$\times$VTBS | L | ^18^ |
| VPARi | interstitial volume in parotid glands | 0.23$\times$MPAR | L | ^25^ |
| VPARv | vascular volume in parotid glands | 0.03$\times$ (1$-$H)$\times$MPAR | L | ^25^ |
| VPROi | interstitial volume in the prostate | 0.25$\times$MPRO | L | ^10^ |
| VPROv | vascular volume in the prostate | 0.004$\times$ (1$-$H)$\times$MPRO | L | ^10^ |
| VRBMv | vascular volume in red bone marrow | 0.04$\times$VTBS | L | ^18^ |
| VRBMi | interstitial volume in red bone marrow | VRBMv$\times$(5.2/1.4) | L | ^12^ |
| VRESv | vascular volume in the remainder, i = all organs | VTBS$-\sum_{i} V_{v,i}$ | L |  |
| VRESi | interstitial volume in the remainder | VRESv$\times$ (831/204) | L | ^12^ |
| VSKIv | vascular volume in skin | 0.03$\times$VTBS | L | ^18^ |
| VSKIi | interstitial volume in skin | VSKIv$\times$ (1125/127) | L | ^12^ |
| VSUBi | interstitial volume in Submandibular glands | 0.23$\times$MSUB | L | ^25^ |
| VSUBv | vascular volume in Submandibular glands | 0.03$\times$ (1$-$H) $\times$MSUB | L | ^25^ |

Table S2. S-values used for calculating absorbed doses in tumours and kidneys

| Variable | Description | S-value [Gy/min/MBq] | Ref. |
| --- | --- | --- | --- |
| $\boldsymbol{S}_{\boldsymbol{K\leftarrow K}}$ | Dose factor kidneys to kidneys | $4.82\times{10}^{-6}\times\frac{0.299}{MKID}$ | ^11^ |
| $\boldsymbol{S}_{\boldsymbol{TU\leftarrow TU}}$ | Dose factor tumour to tumour | $1.38\times{10}^{-3}\times{(MTU\times1000)}^{-1}+2.01\times{10}^{-5}\times{(MTU\times1000)}^{-2/3}-1.83\times{10}^{-5}\times{(MTU\times1000)}^{-1/3}$ | ^26^ |

Figure S2. Biokinetics of [^68^Ga]Ga-PSMA-I&T. Representative biokinetic data and PBPK model-fitted TACs for [^68^Ga]Ga-PSMA-I&T in the kidneys, liver, spleen, tumour 1, tumour 2, and the whole body of a typical patient. The PBPK model is fitted to the measured time–activity data of all organs and tumours simultaneously within a single optimisation process. The organ and tumour time-activity data are derived from quantitative PET/CT imaging. As no direct whole-body measurements were available after the PET/CT image acquisition, the whole-body TAC is estimated using the PBPK model. For the liver and spleen, the early phase of the total-organ TAC reflects the superposition of the blood activity peak following bolus injection and early tissue distribution. In the kidneys, the early vascular contribution is superimposed on rapid renal extraction, glomerular filtration, tubular distribution/retention, and PSMA-mediated uptake. Consequently, liver and spleen activity show a pronounced early distribution phase, whereas renal activity increases more gradually ^27^.

Figure S3. Biokinetics of [^177^Lu]Lu-PSMA-I&T. Representative biokinetic data and PBPK model-fitted TACs for [^177^Lu]Lu-PSMA-I&T in the kidneys, liver, spleen, tumour 1, tumour 2, and the whole body of a typical patient. The PBPK model is fitted to the measured time–activity data of all organs and tumours simultaneously within a single optimisation process. The organ and tumour time-activity data are derived from quantitative SPECT/CT imaging, while whole-body activity measurements were acquired repeatedly during hospitalisation using a dedicated ceiling-mounted probe-based whole-body counting system and incorporated into the model. For the liver and spleen, the early phase of the TACs is less pronounced than after the diagnostic [^68^Ga]Ga-PSMA-I&T administration shown in Figure S2, reflecting the approximately 5-min infusion used for therapeutic administration, which broadens and reduced the early blood activity peak.

Figure S4. Convergence of the calculated total-effect ($S_{i}^{T}$) and main-effect ($S_{i}$) sensitivity indices for the input parameters of the PBPK model, with absorbed doses to the kidneys, tumour 1, tumour 2, and tumour-rest as output variables in the sensitivity analysis. Various sampling sizes were tested to evaluate the convergence behaviour of both sensitivity indices. Only input parameters with $S_{i}^{T}$ and $S_{i}$ values exceeding 0.1 for at least one patient are displayed.

Figure S5. Convergence of absorbed doses (ADs) and their associated standard deviations (SDs) for the kidneys, tumour 1, tumour 2, and tumour-rest in the same patient. The results are shown as a function of increasing sample size to illustrate the stability and precision of the dose estimates derived from the PBPK model.

Figure S6: Histograms of the fifteen patient-specific PBPK model parameters (Table 1) distributions used for the intra-patient GSA in two randomly selected patients, neglecting parameter correlations. Each histogram shows the frequency of occurrence of sampled parameter values. Sampling was performed using the Latin Hypercube Sampling (LHS) method with log-normal parameter distributions and a sample size of 2048. Displaying both patients in the same figure enables direct comparison of inter-patient variability in the parameter distributions.

Table S3: ADs and associated uncertainty metrics (SD, CV and 95% CI width) for kidneys and tumours in all ten patients, calculated using GSA with and without the incorporation of parameter correlations. Statistical analysis with paired Wilcoxon signed-rank tests demonstrated no significant difference in kidney ADs between the two approaches (p = 0.064), whereas a significant reduction in the uncertainty of ADs in kidneys was observed after including parameter correlations (p = 0.002). In contrast, tumour ADs differed significantly between the two GSA approaches, both in their mean values and associated SDs (p < 0.001). Consistently, CVs and 95% CI widths were lower when correlations were incorporated.

| AD $\boldsymbol{\pm}$ SD [Gy] (CV%, 95% CI width) | | | | | |
| --- | --- | --- | --- | --- | --- |
|  | Correlation | Kidneys | Tumour 1 | Tumour 2 | Tumour Rest |
| P1 | No  Yes | 4.4 ± 0.6 (14, 2.4)  4.6 ± 0.5 (10, 1.9) | 18.8 ± 3.5 (19, 13.8)  18.9 ± 3.0 (16, 11.9) | 11.7 ± 2.3 (19, 8.8)  11.7 ± 1.9 (16, 7.3) | 7.2 ± 1.7 (24, 6.7)  7.0 ± 1.3 (19, 5.3) |
| P2 | No  Yes | 4.1 ± 1.2 (28, 4.5)  4.0 ± 0.9 (21, 3.4) | 2.0 ± 1.7 (85, 6.6)  2.0 ± 1.6 (84, 6.5) | 2.9 ± 2.1 (70, 8.1)  2.9 ± 2.0 (69, 7.9) | 2.0 ± 1.4 (69, 5.4)  2.0 ± 1.4 (69, 5.3) |
| P3 | No  Yes | 3.9 ± 0.5 (14, 2.0)  3.8 ± 0.4 (10, 1.4) | 5.2 ± 1.0 (20, 4.1)  5.2 ± 0.8 (16, 3.3) | 3.7 ± 0.8 (23, 3.3)  3.6 ± 0.7 (20, 2.8) | 5.2 ± 1.5 (30, 6.0)  5.2 ± 1.4 (26, 5.4) |
| P4 | No  Yes | 7.3 ± 1.1 (15, 4.2)  7.2 ± 0.8 (11, 3.1) | 18.9 ± 3.3 (17, 12.7)  18.8 ± 3.0 (16, 11.6) | 7.3 ± 1.5 (20, 5.8)  7.2 ± 1.2 (16, 4.7) | 10.3 ± 1.9 (18, 7.3)  10.3 ± 1.9 (19, 7.6) |
| P5 | No  Yes | 5.1 ± 0.6 (11, 2.2)  5.1 ± 0.5 (9, 1.8) | 27.0 ± 4.2 (16, 11.0)  26.8 ± 3.3 (12, 12.9) | 17.6 ± 2.8 (16, 8.6)  17.5 ± 2.2 (13, 8.7) | 4.5 ± 2.2 (49, 34.7)  4.4 ± 2.1 (47, 8.2) |
| P6 | No  Yes | 3.3 ± 0.5 (16, 2.0)  3.3 ± 0.4 (12, 1.5) | 14.5 ± 2.6 (18, 10.2)  14.4 ± 2.2 (16, 8.8) | 3.9 ± 0.8 (20, 3.1)  3.9 ± 0.6 (15, 2.3) | 19.9 ± 5.0 (25, 19.6)  19.9 ± 3.4 (17, 13.2) |
| P7 | No  Yes | 4.0 ± 0.6 (14, 2.2)  4.0 ± 0.5 (12, 1.8) | 6.2 ± 1.3 (20, 4.9)  6.2 ± 1.1 (18, 4.3) | 4.5 ± 0.9 (21, 3.7)  4.5 ± 0.8 (18, 3.1) | 5.5 ± 1.5 (27, 5.8)  5.5 ± 1.3 (24, 5.3) |
| P8 | No  Yes | 6.1 ± 0.8 (12, 2.9)  6.1 ± 0.7 (11, 2.6) | 24.9 ± 4.3 (17, 16.7)  24.6 ± 3.4 (14, 13.4) | 12.7 ± 2.2 (17, 8.6)  12.6 ± 1.7 (14, 6.8) | 15.9 ± 4.8 (30, 18.4)  16.1 ± 4.9 (30, 19.1) |
| P9 | No  Yes | 3.0 ± 0.6 (21, 2.5)  3.0 ± 0.4 (14, 1.7) | 8.2 ± 2.1 (26, 8.3)  8.1 ± 1.3 (16, 5.0) | 11.2 ± 2.8 (25, 11.0)  11.0 ± 1.6 (15, 6.4) | 12.3 ± 3.5 (28, 13.7)  12.2 ± 2.9 (24, 11.4) |
| P10 | No  Yes | 5.6 ± 0.7 (12, 2.7)  5.6 ± 0.5 (9, 2.0) | 9.7 ± 1.8 (19, 7.1)  9.6 ± 1.5 (15, 5.8) | 10.8 ± 2.0 (18, 7.8)  10.7 ± 1.7 (15, 6.5) | 8.6 ± 2.9 (34, 11.5)  8.5 ± 2.7 (31, 10.5) |

AD: absorbed dose; SD: standard deviation; CV: coefficient of variation; CI: confidence interval;
GSA: global sensitivity analysis

Table S4: ADs and associated uncertainty metrics (SD, CV and 95 % CI width) for liver and spleen in the ten-patient cohort, calculated using GSA with and without the incorporation of parameter correlations. While the estimated ADs for both the liver and spleen remained unchanged between the two GSA approaches, incorporating parameter correlations resulted in lower uncertainty, CV, and 95 % CI width for the estimated ADs in both organs.

|  | Parameter Correlations | AD [Gy]  median [min – max] | SD [Gy]  median [min – max] | CV [%]  median [min – max] | 95 % CI width  median [min – max] |
| --- | --- | --- | --- | --- | --- |
| Liver | Yes | 0.25 [0.20 – 0.45] | 0.03 [0.02 – 0.08] | 10 [9 – 25] | 0.23 [0.07 – 0.80] |
| Liver | No | 0.25 [0.20 – 0.45] | 0.03 [0.02 – 0.11] | 13 [11 – 32] | 0.34 [0.23 – 1.08] |
| Spleen | Yes | 0.39 [0.19 – 0.75] | 0.05 [0.02 – 0.09] | 11 [9 – 29] | 0.52 [0.12 – 1.06] |
| Spleen | No | 0.39 [0.19 – 0.75] | 0.07 [0.03 – 0.11] | 14 [11 – 34] | 0.82 [0.33 – 1.31] |

AD: absorbed dose; SD: standard deviation; CV: coefficient of variation; CI: confidence interval; GSA: global sensitivity analysis.

|  |  |
| --- | --- |

Figure S7. Bland-Altman analysis of the agreement between two GSA, with and without parameter correlation, approaches for AD uncertainty estimation in (left) kidney and (right) tumour. Solid red lines indicate the mean difference, with dashed black lines representing the 95% limits of agreement. The kidney shows a consistent negative bias with relatively uniform scatter; however, interpretation is limited by the small sample size (n = 10). In contrast, the tumour exhibits a larger systematic offset with a negative proportional trend, differences become increasingly negative as mean uncertainty increases, indicating that the two GSA approaches diverge more substantially at higher uncertainty magnitudes. This suggests poorer and more variable agreement for tumour AD uncertainty estimation, with disagreement that scales with the magnitude of uncertainty itself.

**References**

1.	Winter G, Drescher, A., Baur, B., Solbach, C., Reske, S. N., Beer, A. J. Comparative analysis of chelator-modified peptides for imaging of prostate carcinoma. In: *Annual Congress EANM*. Suppl 2. 41; 2014:S414 (P200).

2.	Kletting P, Schuchardt C, Kulkarni HR, et al. Investigating the Effect of Ligand Amount and Injected Therapeutic Activity: A Simulation Study for 177Lu-Labeled PSMA-Targeting Peptides. *PLOS ONE*. 2016;11(9):e0162303. doi:10.1371/journal.pone.0162303

3.	Begum NJ, Thieme A, Eberhardt N, et al. The Effect of Total Tumor Volume on the Biologically Effective Dose to Tumor and Kidneys for 177Lu-Labeled PSMA Peptides. *J Nucl Med*. 2018;59(6):929-933. doi:10.2967/jnumed.117.203505

4.	McCutchan EA. Nuclear Data Sheets for A = 68. *Nucl Data Sheets*. 2012;113(6):1735-1870. doi:10.1016/j.nds.2012.06.002

5.	Kondev FG. Nuclear Data Sheets for A=177. *Nucl Data Sheets*. 2019;159:1-412. doi:10.1016/j.nds.2019.100514

6.	Du Bois D, Du Bois EF. A formula to estimate the approximate surface area if hight and weight be known. *Arch Intern Med*. 1916;XVII(6_2):863-871. doi:10.1001/archinte.1916.00080130010002

7.	Leggett RW, Williams LR. A proposed blood circulation model for Reference Man. *Health Phys*. 1995;69(2):187-201. doi:10.1097/00004032-199508000-00003

8.	Kletting P, Kull T, Maaß C, et al. Optimized Peptide Amount and Activity for ^90^Y-Labeled DOTATATE Therapy. *J Nucl Med Off Publ Soc Nucl Med*. 2016;57(4):503-508. doi:10.2967/jnumed.115.164699

9.	Łuczyńska E, Heinze-Paluchowska S, Blecharz P, et al. Correlation between CT Perfusion and Clinico-Pathological Features in Prostate Cancer: A Prospective Study. *Med Sci Monit Int Med J Exp Clin Res*. 2015;21:153-162. doi:10.12659/MSM.891401

10.	Buckley DL, Roberts C, Parker GJM, Logue JP, Hutchinson CE. Prostate cancer: evaluation of vascular characteristics with dynamic contrast-enhanced T1-weighted MR imaging--initial experience. *Radiology*. 2004;233(3):709-715. doi:10.1148/radiol.2333032098

11.	Stabin MG, Sparks RB, Crowe E. OLINDA/EXM: the second-generation personal computer software for internal dose assessment in nuclear medicine. *J Nucl Med*. 2005;46(6):1023-1027.

12.	Shah DK, Betts AM. Towards a platform PBPK model to characterize the plasma and tissue disposition of monoclonal antibodies in preclinical species and human. *J Pharmacokinet Pharmacodyn*. 2012;39(1):67-86. doi:10.1007/s10928-011-9232-2

13.	Kletting P, Thieme A, Eberhardt N, et al. Modeling and Predicting Tumor Response in Radioligand Therapy. *J Nucl Med Off Publ Soc Nucl Med*. 2019;60(1):65-70. doi:10.2967/jnumed.118.210377

14.	Hollenberg NK, Adams DF, Solomon HS, Rashid A, Abrams HL, Merrill JP. Senescence and the renal vasculature in normal man. *Circ Res*. 1974;34(3):309-316. doi:10.1161/01.res.34.3.309

15.	Fresco GF, DiGiorgio F, Curti GL. Simultaneous estimation of glomerular filtration rate and renal plasma flow. *J Nucl Med Off Publ Soc Nucl Med*. 1995;36(9):1701-1706.

16.	Schmidt MM, Wittrup KD. A modeling analysis of the effects of molecular size and binding affinity on tumor targeting. *Mol Cancer Ther*. 2009;8(10):2861-2871. doi:10.1158/1535-7163.MCT-09-0195

17.	Groothuis DR. The blood-brain and blood-tumor barriers: A review of strategies for increasing drug delivery. *Neuro-Oncol*. 2000;2(1):45-59. doi:10.1093/neuonc/2.1.45

18.	International commission on radiological protection (ICPR). Basic anatomical and physiological data for use in radiological protection: reference values. A report of age- and gender-related differences in the anatomical and physiological characteristics of reference individuals. ICRP Publication 89. *Ann ICRP*. 2002;32(3-4):5-265.

19.	Snyder, W. S., Cook, M. J., Nasset, E. S., Karhausen, L. R., Howells, G. P., Tipton, I. H. *Reference Man: Anatomical, Physiological and Metabolic Characteristics. Report of the Task Group on Reference Man*. Elsevier, New York:354.

20.	Bingham CM, Castro A, Realini T, Nguyen J, Hogg JP, Sivak-Callcott JA. Calculated CT Volumes of Lacrimal Glands in Normal Caucasian Orbits. *Ophthal Plast Reconstr Surg*. 2013;29(3):157. doi:10.1097/IOP.0b013e3182859751

21.	International commission on radiological protection (ICPR). *Report of the Task Group on Reference Man. ICRP Publication 23*. Pergamon Press, Oxford; 1975.

22.	Rumboldt Z, Al-Okaili R, Deveikis JP. Perfusion CT for head and neck tumors: pilot study. *AJNR Am J Neuroradiol*. 2005;26(5):1178-1185.

23.	O’Keefe DS, Bacich DJ, Heston WDW. Comparative analysis of prostate-specific membrane antigen (PSMA) versus a prostate-specific membrane antigen-like gene. *The Prostate*. 2004;58(2):200-210. doi:10.1002/pros.10319

24.	Ben Jemaa A, Bouraoui Y, Sallami S, et al. Co-expression and impact of prostate specific membrane antigen and prostate specific antigen in prostatic pathologies. *J Exp Clin Cancer Res CR*. 2010;29(1):171. doi:10.1186/1756-9966-29-171

25.	Berggreen E, Wiig H. Lowering of interstitial fluid pressure in rat submandibular gland: a novel mechanism in saliva secretion. *Am J Physiol Heart Circ Physiol*. 2006;290(4):H1460-1468. doi:10.1152/ajpheart.00887.2005

26.	Stabin MG, Siegel JA. Physical models and dose factors for use in internal dose assessment. *Health Phys*. 2003;85(3):294-310. doi:10.1097/00004032-200309000-00006

27.	Herrmann K, Bluemel C, Weineisen M, et al. Biodistribution and Radiation Dosimetry for a Probe Targeting Prostate-Specific Membrane Antigen for Imaging and Therapy. *J Nucl Med Off Publ Soc Nucl Med*. 2015;56(6):855-861. doi:10.2967/jnumed.115.156133
